# Diverse Cyclization Pathways Between Nitriles with Active *α*-Methylene Group and Ambiphilic 2-Pyridylselenyl Reagents Enabled by Reversible Covalent Bonding

**DOI:** 10.3390/ijms252312798

**Published:** 2024-11-28

**Authors:** Alexey A. Artemjev, Alexander A. Sapronov, Alexey S. Kubasov, Alexander S. Peregudov, Alexander S. Novikov, Anton R. Egorov, Victor N. Khrustalev, Alexander V. Borisov, Zhanna V. Matsulevich, Namiq G. Shikhaliyev, Valentine G. Nenajdenko, Rosa M. Gomila, Antonio Frontera, Andreii S. Kritchenkov, Alexander G. Tskhovrebov

**Affiliations:** 1 Research Institute of Chemistry, Peoples’ Friendship University of Russia, 6 Miklukho-Maklaya Street, 117198 Moscow, Russia; 2 Kurnakov Institute of General and Inorganic Chemistry, 31 Leninsky Prospekt, 119991 Moscow, Russia; 3 Nesmeyanov Institute of Organoelement Compounds, Russian Academy of Sciences, 119334 Moscow, Russia; 4 N.D. Zelinsky Institute of Organic Chemistry, Russian Academy of Sciences, Leninsky Prospekt 47, 119334 Moscow, Russia; 5 Department of Chemistry, R.E. Alekseev Nizhny Novgorod State Technical University, Minin Street, 24, 603155 Nizhny Novgorod, Russia; 6 Department of Chemical Engineering, Baku Engineering University, Hasan Aliyev Street 120, Baku AZ0101, Azerbaijan; 7 Department of Chemistry, M.V. Lomonosov Moscow State University, 1 Leninskie Gory, 119991 Moscow, Russia; 8 Departament de Química, Universitat de les Illes Balears, 07122 Palma de Mallorca, Spain; 9 Branch of Petersburg Nuclear Physics Institute Named by B.P. Konstantinov of National Research Centre «Kurchatov Institute», Institute of Macromolecular Compounds, Bolshoi pr. VO 31, 199004 St. Petersburg, Russia

**Keywords:** selenazoles, nitriles, non-covalent interactions, halogen bonding, chalcogen bonding

## Abstract

Herein, we describe a novel coupling between ambiphilic 2-pyridylselenyl reagents and nitriles featuring an active α-methylene group. Depending on the solvent employed, this reaction can yield two distinct types of cationic pyridinium-fused selenium-containing heterocycles, 1,3-selenazolium or 1,2,4-selenadiazolium salts, in high yields. This is in contrast to what we observed before for other nitriles. Notably, the formation of selenadiazolium is reversible, gradually converting into the more thermodynamically stable selenazolium product in solution. Our findings reveal, for the first time, the reversible nature of 1,3-dipolar cyclization between the CN triple bond and 2-pyridylselenyl reagents. Nitrile substitution experiments in the adducts confirmed the dynamic nature of this cyclization, indicating potential applications in dynamic covalent chemistry. DFT calculations revealed the mechanistic pathways for new cyclizations, suggesting a concerted [3 + 2] cycloaddition for the formation of selenadiazolium rings and a stepwise mechanism involving a ketenimine intermediate for the formation of selenazolium rings. Natural bond orbital analysis confirmed the involvement of σ-hole interactions and lone pair to σ* electron donation in these processes. Additionally, theoretical investigations of σ-hole interactions were performed, focusing on the selenium-centered contacts within the new compounds.

## 1. Introduction

Nitriles are attractive synthons for the construction of diverse nitrogen-containing heterocycles which, in turn, are of great importance in medicinal, organic chemistry, and material science [1,2,3]. Cycloaddition reactions involving nitriles and 1,3-dipoles provide a highly efficient atom- and step-economic method for synthesizing nitrogen-containing five-membered heterocycles [4]. Currently, numerous significant dipolar cycloaddition (DCA) reactions have been documented, including the coupling of nitriles with azides, nitrones, nitrile oxides, as well as other nitrilium and diazonium betaines reported in the literature [5,6,7,8]. In contrast, pathways to form bonds between main group elements (except for nitrogen and carbon) and to directly access products containing these elements are poorly developed [9].

Moreover, utilization of nitriles in the synthesis is frequently hindered by their inertness. Efficient electrophilic or nucleophilic activation of nitriles can be achieved by coordinating them to electron-poor, high-oxidation-state metal centers or electron-rich, low-valent metal centers, respectively [10,11,12,13].

**Scheme 1:**
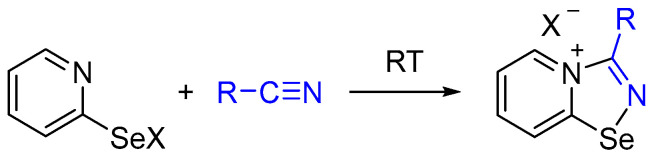
1,3-Dipolar cyclisation reactions of nitriles with ambiphilic 2-pyridylselenyl reagents.

Activation of nitriles with organometalloid compounds can be advantageous because the activator of the CN triple bond is inherently present in the substrate. We recently reported a novel cyclization reaction involving ambiphilic 2-pyridylselenyl reagents and a broad range of substrates containing CN triple bonds (Scheme 1) [14,15,16,17,18,19,20,21,22,23].

This reaction stands out starkly from other DCA reactions involving nitriles reported to date, as it proceeds under mild conditions without the need for additional nitrile activation. Arguably, the metalloid nature of the selenium center itself can activate nitriles, akin to transition metals in this regard [24]. Importantly, the reaction is effective for a broad scope of RCN substrates, where R can be alkyl aryl, heteroaryl, Br, CCl_3_, NR′_2_, SR′, SeR′, and even for NaN(CN)_2_, thereby enabling the synthesis of novel Se-containing heterocycles. This is significant because organoselenium compounds hold considerable pharmacological importance [25,26].

Here, we describe reactions of 2-pyridylselenyl reagents with nitriles containing the so-called active α-methylene group. We demonstrate that the reaction outcome depends on the conditions employed, allowing the facile preparation of Se-containing heterocyclic scaffolds, which are inaccessible by the other known methods. Furthermore, in this study, we demonstrate for the first time that the addition of 2-pyridylselenyl reagents to the CN triple bond is reversible: the nitrile in its adduct with a 2-pyridylselenyl reagent can be replaced by another nitrile.

It is worth noting that the well-established dynamic covalent nature of Se−X (Se−Se, Se−N) bonds has been successfully utilized in polymer systems and supramolecular chemistry [27,28,29]. In this context, our findings pave the way for new opportunities in this area of research.

## 2. Results and Discussion

The study described herein is a part of our systematic investigation of the reactivity of ambiphilic 2-pyridylselenyl reagents towards unsaturated substrates [14,15,16,17,18,19,20,21,22,23,30]. Having initially confirmed that both alkyl and aryl nitriles undergo 1,3-dipolar cycloaddition (DCA) with 2-pyridylselenyl reagents, our objective was to extend this reaction to encompass nitriles containing the so-called active α-methylene group.

**Scheme 2:**
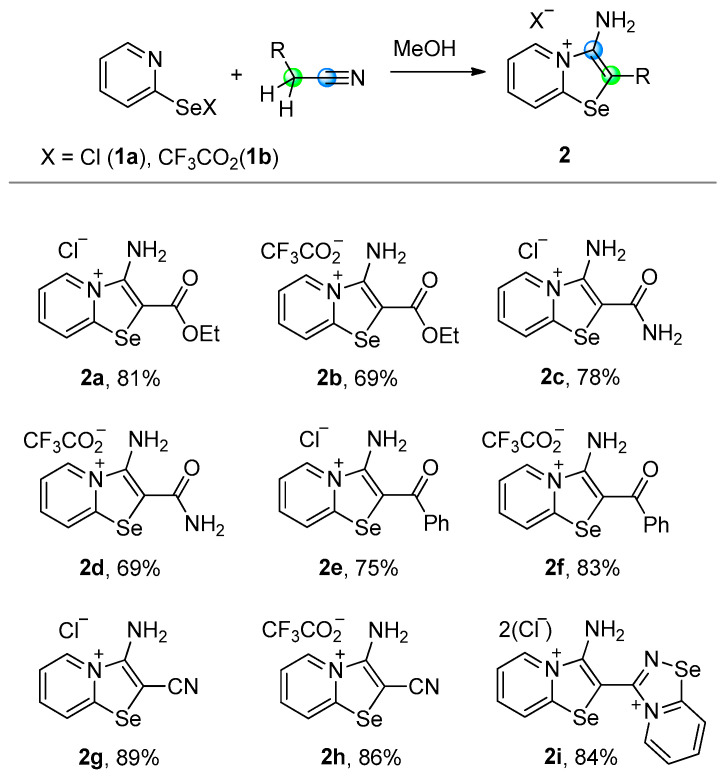
Synthesis of **2a**–**2i**.

When a solution of 2-pyridylselenyl chloride (**1a**) was stirred with an excess of ethylcyanoacetate in MeOH for 24 h, gradual formation of a bright-yellow precipitate **2a** took place (Scheme 2). The bright color of the solid already indicated a distinct reaction outcome compared to the previously investigated nitriles, which produced colorless adducts.

Isolation (81% yield) and analysis of **2a** by NMR spectroscopy, mass spectrometry, and elemental analysis suggested the formation of 1:1 adduct between **1a** and ethylcyanoacetate. However, the ^77^Se NMR spectrum of **2a** (a singlet at 584.0 ppm) was inconsistent with the 1,2,4-selenadiazolium structure. Surprisingly, crystallographic analysis (see below) unveiled that **2a** was unprecedented bicyclic pyridine-fused 1,3-selenazolium chloride (Scheme 2). In a similar way, 2-pyridylselenyl trifluoroacetate (**1b**) reacted with ethylcyanoacetate to produce **2b** (Scheme 2). Employing a similar procedure, **1a** and **1b** could be coupled with 2-cyanoacetamide, benzoylacetonitrile, malononitrile, and even nitrile **3b** (for its synthesis see further), yielding pyridine-fused selenazolium salts **2c**–**2i** in good to excellent yields (Scheme 2).

**Scheme 3:**
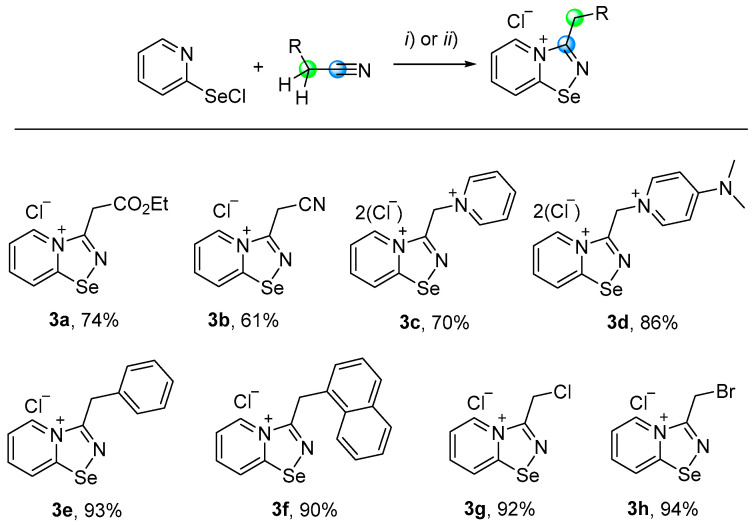
Synthesis of **3a**–**3h**. (***i***) Et_2_O for **3a** and **3b**, (***ii***) Et_2_O or MeOH for **3c**–**3h**.

Interestingly, when the reactions between **1a** and ethylcyanoacetate or malononitrile were performed in Et_2_O, 1,2,4-selenadiazolium salts **3a** and **3b** were isolated in high yields (Scheme 3).

Next, we examined the reactions of nitriles containing *N*-pyridinium, *N*-(*p*-Me_2_N)-pyridinium, Ph, Napth, Cl, and Br substituents adjacent to the methylene group. In all cases, we observed the exclusive formation of selenadiazolium salts **3c**–**3h**, resulting from the addition to the CN triple bond (Scheme 3, experimental part). Notably, this outcome was independent of the solvent used. Arguably, nucleophilicity of the *α*-CH_2_R carbon atom attached to the *N*-pyridinium Ph, Napth, Cl, or Br substituents is not sufficient for the formation of selenazolium product.

**Figure 1:**
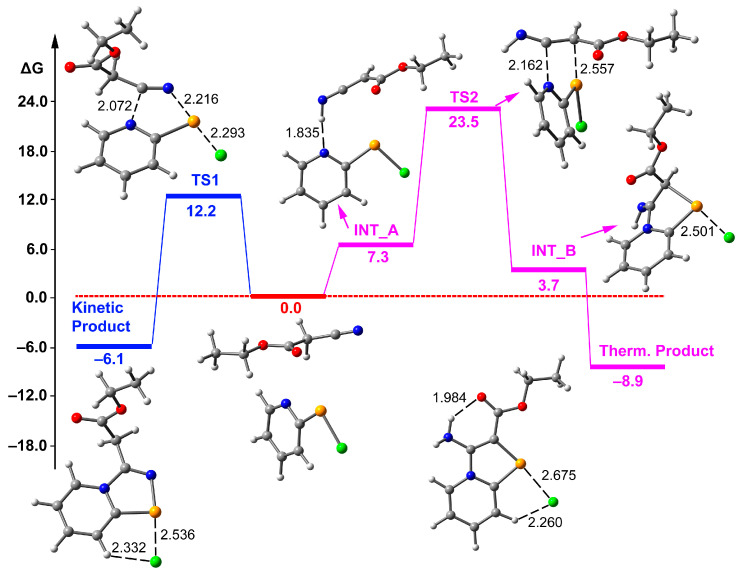
Energetic profile (PBE0-D4/def2-TZVP level of theory) for the transformation of **1a** into selenadiazolium (blue path) of selenazolium (fuchsia path) derivatives. Energies are indicated in kcal/mol.

To elucidate the reaction mechanism, we conducted DFT calculations at the PBE0-D4/def2-TZVP level of theory. As the exemplifying reaction, we have used 2-pyridylselenyl chloride and ethylcyanoacetate and initially analyzed the formation of selenadiazolium cation through 1,3-dipolar cycloaddition (depicted as the blue path in Figure 1). The polar 1,3-cycloaddition has a relatively low barrier (12.2 kcal/mol) and proceeds via a concerted transition state, resulting in the formation of a selenadiazolium ring. During this process, the chloride anion remains in close proximity to the Se atom, establishing a short chalcogen bond (2.536 Å). The reaction is exergonic (with a ΔG of −6.1 kcal/mol) and likely reversible since the step barrier of the reverse reaction is 18.3 kcal/mol.

Regarding the selenazolium product, we propose a mechanism involving the formation of a transient intermediate. This intermediate takes the form of the EWG–CH=C=NH ketenimine tautomer that is stabilized by a H-bond with the N-atom of pyridine (see INT_A in the fuchsia path in Figure 1). The enhanced acidity of the H-atoms in the nitriles used in this work facilitates the formation of the tautomer. Moreover, the formation of this tautomeric species is likely facilitated by the protic solvent used in the experiments (MeOH), which can assist in the migration of the hydrogen atom from the CH_2_ group to the nitrogen atom. This intermediate is 7.3 kcal/mol higher in energy than the starting material. Then, a 3 + 2 cycloaddition reaction occurs, with a step barrier of 16.2 kcal/mol, and a global energy barrier of 23.5 kcal/mol occurs, yielding a second intermediate (INT-B). It should be mentioned that 3 + 2 cycloaddition reactions involving ketenimines have been reported in the literature [31]. The cycloaddition product (INT-B) undergoes a second tautomerization to yield the final product (selenazolium), which is thermodynamically more stable than the kinetic one (selenadiazolium). This path is irreversible, since the global inverse barrier is 32.4 kcal/mol. It can be observed that the selenazolium cation is stabilized by an intramolecular H-bond that is formed by the amino group. This intramolecular H-bond is indeed important in determining the thermodynamic product. In fact, if R = Ph is used for the calculations, the energy difference between both products is reduced to 0.32 kcal/mol where the selenadiazolium product is more stable, in line with the formation of compound **3e** in high yields.

**Scheme 4:**
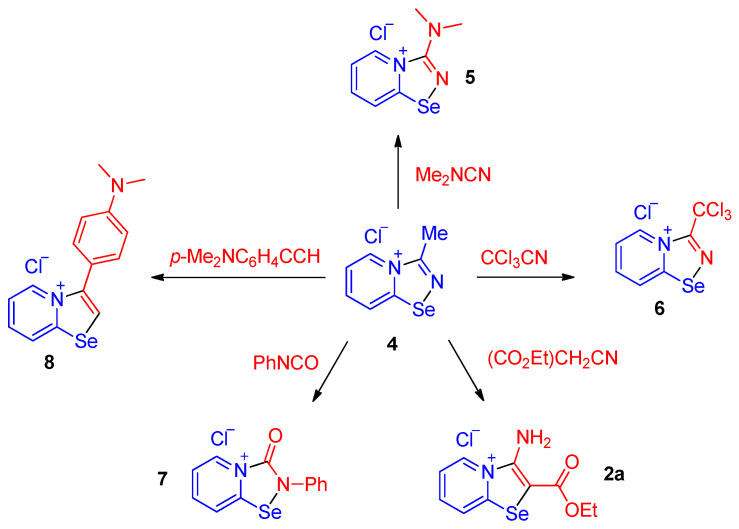
Caption. Synthesis of **5**–**8**, **2a**.

In order to support the proposed mechanism, we performed several additional experiments. First, we demonstrated that the C≡N group binding by 2-pyridylselenyl chloride is reversible. The adduct of 2-pyridylselenyl chloride and acetonitrile **4** reacts with an excess of other nitriles (we employed either CCl_3_CN or Me_2_NCN as two extreme cases; Scheme 4), resulting in the formation of corresponding nitrile adducts **5** and **6**.

Ethylcyanoacetate, phenylisocyanate, and *p*-dimethylaminophenylacetylene also substitute acetonitrile in the adduct, producing **2a**, **7**, and **8**, respectively, in high yields (Scheme 4). These substitution experiments are important because they showcase that covalent fixation of nitriles by bifunctional 2-pyridylselenyl reagents is reversible, supporting the proposed mechanism depicted in Figure 1.

In order to obtain more information about the coupling between **1a** and the active α-methylene group, we performed NMR monitoring experiments. The reaction between **1a** and a 10-fold excess of malononitrile in CD_3_OD resulted in a gradual transformation of **1a** into **2g** (thermodynamic control), along with a minor amount of its isomer **3b** (kinetic control) within ca. 1 h (Figure S1). A similar picture was observed for the reaction between **1a** and an excess of ethylcyanoacetate (Figure S2). Interestingly, pure **3a** gradually isomerized into thermodynamically more favorable **2a** in CD_3_OD (Figure S3). These were all indicated on the reversible covalent bonding in the adducts between 2-pyridylselenylchloride and nitriles.

**Figure 2:**
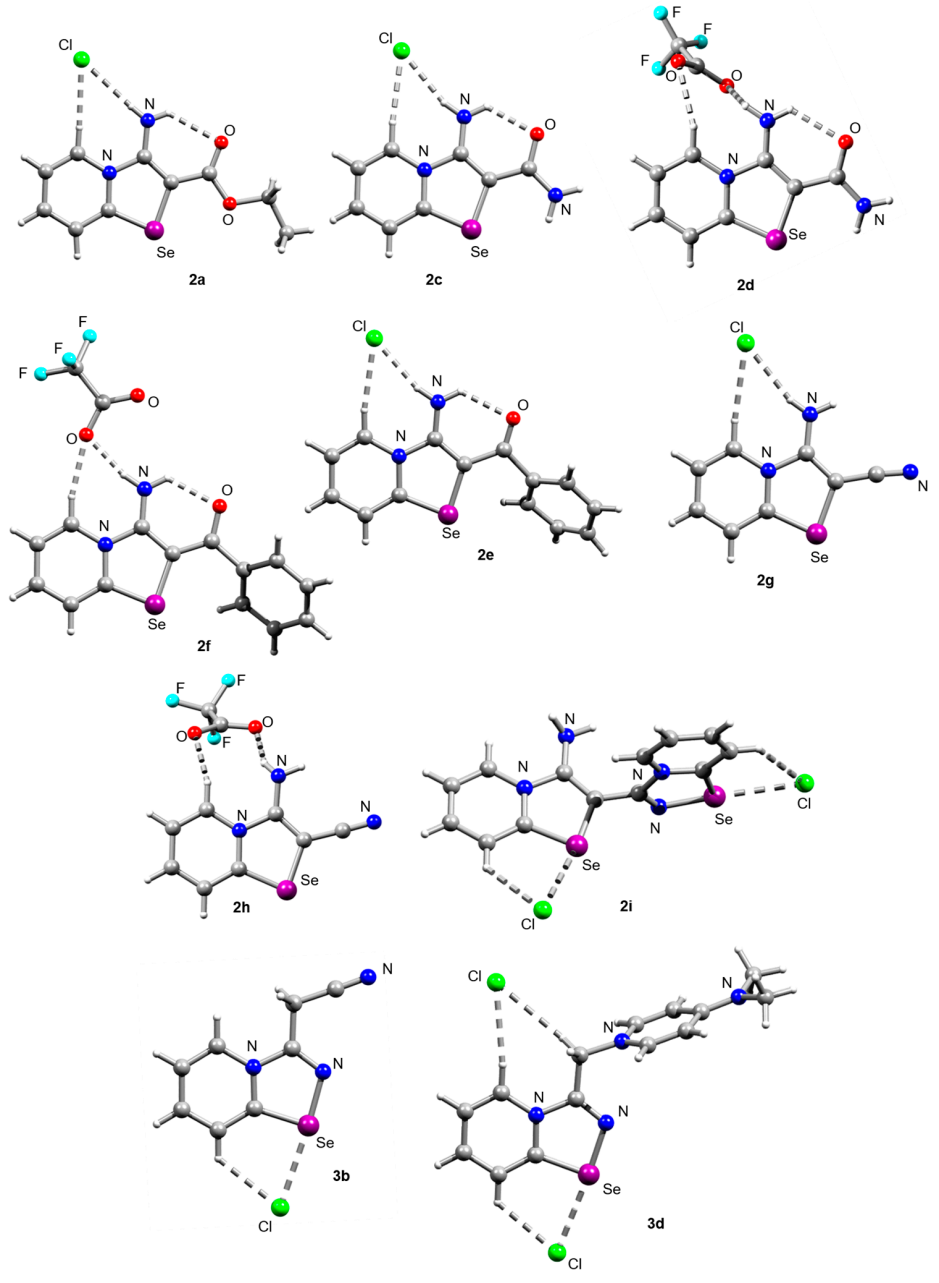
Ball-and-stick representations of crystal structures of **2a**, **2c**–**2i**, **3b**, and **3d**. Grey and light-grey spheres represent carbon and hydrogen, respectively.

Compounds **2a**, **2c**–**2i**, **3b**, and **3d** could be recrystallized from the corresponding solvent (for details, see the SI) to yield single crystals, suitable for analysis by single-crystal X-ray crystallography, which unveiled the formation of corresponding adducts (Figure 2).

In **2a**, **2c**–**2i**, **3b**, and **3d**, selenium centers adopt a T-shaped geometry (C_py_–Se–C angles are 85.74° (**2a**), 85.85° (**2c**), 85.88° (**2d**), 85.75° (**2e**), 86.47° (**2f**), 84.85° (**2g**), 85.08° (**2h**), and 84.71°(**2i**), and N–Se–C angles are 88.11° (**2i**), 87.12° (**3b**), and 87.15° (**3d**)). The distances C_py_–Se (1.842–1.858 Å), Se–C_sp2_ (1.873–1.900 Å), and C_sp2_=C_sp2_ (1.348–1.390 Å) found for 1,3-selenazolium moieties and C_py_–Se (1.870–1.873 Å), Se–N (1.839–1.853 Å), and C_sp2_=N (1.271–1.276 Å) for—1,2,4-selenadiazolium moieties are within typical ranges for these bonds. Overall, the metrical parameters in **2a**, **2c**–**2i**, **3b**, and **3d** are consistent with typical values found in similar heterocyclic systems [14,15,16,17,18,19,21,30,32,33,34,35,36].

1,2,4-Selenadiazolium cations, formed through the [3 + 2] cycloaddition [22] between 2-pyridylselenyl reagents and nitriles, have demonstrated a pronounced affinity for participating in various ChB interactions [15,16,20,21,37,38]. The chalcogen bonding, a subclass of “σ-hole interactions” [39], has been recently defined by the IUPAC [37]. It is an important noncovalent force in several fields, like catalysis [40], material science [41], biological [42] and medicinal chemistry [43], and supramolecular chemistry [44].

**Figure 3:**
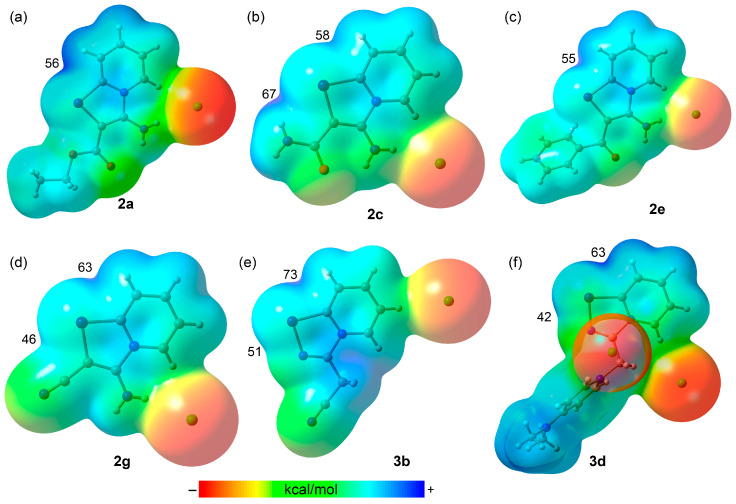
MEP surfaces of compounds **2a** (**a**), **2c** (**b**), **2e** (**c**), **2g** (**d**), **3b** (**e**), and **3d** (**f**). Energies in kcal/mol. Isovalue 0.001 a.u.

The 1,3-selenazolium frameworks described here also contain electron-deficient Se atoms, providing two σ-holes along the extension of the covalent bond axes. To investigate the presence and characteristics of noncovalent interactions in compounds **2a**, **2c**–**2i**, **3b**, and **3d**, and to compare the σ-hole depth of Se in 1,3-selenazolium and 1,2,4-selenadiazolium, we conducted DFT calculations. MEP surface analysis was initially performed to explore the existence and intensity of the σ-holes at the Se atoms, as shown in Figure 3. We focused the analysis on a representative set of compounds to avoid repetition and maintain simplicity.

For compounds **2a** and **2c**, only one σ-hole (local maximum) at the Se is observed, due to the influence of the O-atom in **2a** or the π-system in **2e**. Thus, only one σ-hole is accessible in these compounds. For the remaining compounds, MEP surface analysis reveals the existence of two σ-holes at Se. In all complexes, the most positive σ-hole is influenced by an adjacent C–H or N–H bond, which enhances the positive MEP value.

The calculations included the counterion to ensure neutral systems, better representing the real situation in the solid state. For the 1,3-selenazolium salts, the σ-hole values vary from 46 to 63 kcal/mol, while for 1,2,4-selenadiazolium **3b**, the σ-hole is more intense (73 kcal/mol), indicating that the latter are better chalcogen bond donor molecules. Finally, for the dicationic compound **3d**, where two counterions were used, the MEP values are comparable to the 1,3-selenazolium salt **2g**.

**Figure 4:**
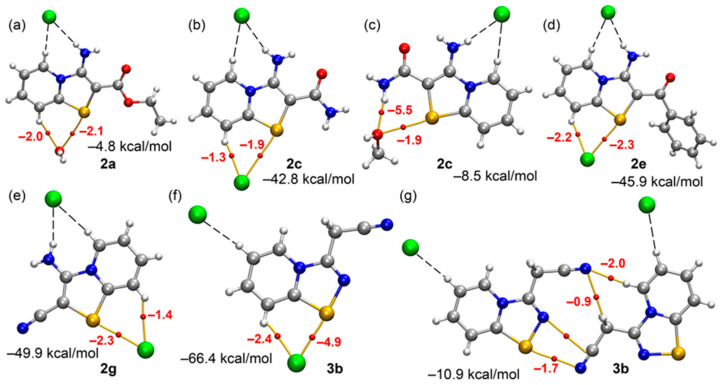
QTAIM analysis of compounds **2a** (**a**), **2c** (**b**,**c**), **2e** (**d**), **2g** (**e**), and **3b** (**f**,**g**). Bond critical points (BCPs) as red spheres and bond paths as orange lines. The individual energies derived from the Vg values for the HBs and ChBs are shown in red close to the BCPs.

We computed the interaction energies for several 1,3-selenazolium chloride ion pairs with both neutral (**2a**, **2c**; Figure 4a,c) and charged (**2c**, **2e**, **2g**; Figure 4b,d,e) electron donors, as well as a 1,2,4-selenadiazolium chloride ion pair with an anion (**3b**; Figure 4f) or another ion pair (homodimer in **3b**; Figure 4g). For the 1,3-selenazolium salts, interactions with neutral donors were moderately strong (−4.8 kcal/mol for water and −8.5 kcal/mol for methanol) and strong with the anion (ranging from −42.8 to −49.9 kcal/mol).

The QTAIM analysis reveals that in all cases, the electron donor atom is linked to the cation by two bond critical points (BCPs, small red spheres) and bond paths (orange lines). The bond paths connect the O or Cl atom to the Se (ChB) and to the H-atom either belonging to the aromatic ring or the amido group (in **2c**; Figure 4c). In the latter case, Se establishes two ChBs, one with the chloride anion and the other with the methanol molecule.

To investigate the relative contributions of ChB and HB, as well as the strengths of both interactions without the strong Coulombic contribution (ion-pair), we estimated the interaction strength using the potential energy density at the BCPs and the method proposed in the literature [45,46]. These values are shown in red in Figure 4. It can be observed that the ChB is slightly stronger than the HB, except in compound **2c** (interaction with MeOH; Figure 4c), where the amido group acts as an H-bond donor.

For the 1,2,4-selenadiazolium compound **3b**, the ChB interaction is the strongest one (−4.9 kcal/mol), consistent with the MEP surface analysis. In the case of the ion pair dimer represented in Figure 4g, the interaction energy is −10.9 kcal/mol, revealing an intricate combination of interactions, as disclosed by the QTAIM analysis. This includes a weak Se···N ChB (−1.7 kcal/mol), two CH···N contacts, and an interesting N···CN interaction.

**Figure 5:**
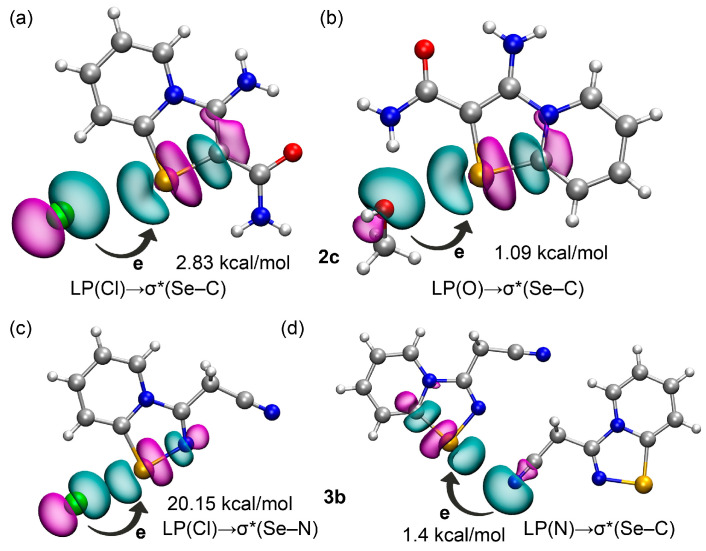
Natural bond orbitals involved in the LP→σ* electron donations in compounds **2c** (**a**,**b**) and **3b** (**c**,**d**). The second-order stabilization energies are indicated.

Finally, a natural bond orbital (NBO) analysis was performed on four compounds to characterize the ChB interactions from an orbital perspective. We selected examples representing all four types of ChBs observed in the compounds, based on the electron donor (Cl, N, and O) and the electron acceptor (Se–C or Se–N bond). The results, shown in Figure 5, evidence the typical LP→σ* electron donation.

For compound **2c**, we examined both σ-hole interactions (Se···Cl and Se···O), revealing that the stabilization energy from the LP(Cl)→σ*(Se–C) charge transfer is larger than that from the LP(O)→σ*(Se–C). In the case of the selenadiazolium **3b**, the LP(Cl)→σ*(Se–N) stabilization is significant, consistent with the strong binding energy (−66.4 kcal/mol; see Figure 4f) and short Cl···Se distance (2.869 Å). For the homodimer of **3b** (Figure 5d), the LP(N)→σ*(Se–C) stabilization is modest (1.4 kcal/mol), in line with the less favorable directionality of the LP→σ* electron donation and the longer N···Se distance (3.170 Å).

It should be noted that the contributions based on the charge-transfer and geometric viewpoints were important in creating the modern ChB concept [47,48,49,50,51,52,53,54,55,56,57,58,59,60,61,62,63,64,65,66].

## 3. Methods

### 3.1. Experimental Details

General remarks. No uncommon hazards derived from the experimental work carried out are noted. All manipulations were carried out in air. All the reagents used in this study were obtained from commercial sources (Aldrich, TCI-Europe, Strem, ABCR). Commercially available solvents were purified by conventional methods and distilled immediately prior to use. Mass spectra were recorded on a Bruker micrOTOF spectrometer equipped with an electrospray ionization (ESI) source; MeOH or MeCN was used as a solvent. NMR spectra were recorded on a Bruker Avance neo 700; chemical shifts (*δ*) are stated in ppm, coupling constants (*J*) in Hz. IR spectra were recorded on a Shimadzu Inspirit IR-Fourier spectrometer, equipped with the QATAR-Singapore attenuated total reflectance sample holder.

Synthetic part.

Synthesis of **2a**–**2i**.

Method A. The respective nitrile (1 equiv.) in MeOH was added to a suspension of 2-pyridylselenyl chloride (1 equiv.), and the resulting mixture was stirred at 50 °C for 6 h. Then, the reaction mixture was evaporated under vacuum. The formed solid was washed with CH_2_Cl_2_ (1 mL), Et_2_O (3 × 3 mL) and dried under vacuum.

Method B. A solution of PIFA (1 equiv.) in MeOH was added dropwise to a solution of 2,2′-Py_2_Se_2_ (1 equiv.) in MeOH. The reaction mixture was kept without stirring for 3 h. Then, the corresponding nitrile (1 equiv.) was added, and the resulting mixture was stirred at RT for 3 h. The reaction mixture was evaporated, washed with Et_2_O (3 × 3 mL), and dried under vacuum.

Method C. 2-Pyridylselenyl chloride (1 equiv.) was added to the respective nitrile in Et_2_O (5 mL), and the resulting mixture was stirred at RT for 6 h. The precipitate gradually formed during the reaction, which was then filtered, washed with Et_2_O (3 × 3 mL), and dried under vacuum.

Compound **2a** was prepared according to Method A. 2-Pyridylselenyl chloride (50 mg, 0.26 mmol) and ethylcyanoacetate (31 mg, 0.26 mmol) were used. Yield: 58 mg (73%). ^1^H NMR (700 MHz, D_2_O) *δ* 9.15 (d, *J* = 6.9 Hz, 1H), 8.59 (d, *J* = 8.7 Hz, 1H), 8.24 (t, *J* = 8.0 Hz, 1H), 7.97 (t, *J* = 7.1 Hz, 1H), 4.44 (q, *J* = 7.1 Hz, 2H), 1.39 (t, *J* = 7.1 Hz, 3H). ^13^C{^1^H} NMR (176 MHz, D_2_O) *δ* 164.6, 156.2, 147.7, 137.8, 134.2, 127.6, 122.5, 96.2, 63.2, 13.4. ^77^Se NMR (95 MHz, MeOH-*d*_4_) *δ* 584.0. MS (ESI^+^), found: 270.9983 [M − Cl]^+^; calcd. for C_10_H_11_N_2_O_2_Se (^80^Se): 270.9981.

Compound **2b** was prepared according to Method B. 2,2′-Py_2_Se_2_ (46 mg, 0.15 mmol), ethylcyanoacetate (156 µL, 1.46 mmol), and PIFA (63 mg, 0.15 mmol) were used. Yield: 77 mg (69%). ^1^H NMR (700 MHz, D_2_O) *δ* 9.13 (dt, *J* = 6.9, 1.0 Hz, 1H), 8.57 (dt, *J* = 8.7, 1.1 Hz, 1H), 8.23 (td, *J* = 8.6, 7.3, Hz, 1H), 7.95 (td, *J* = 7.1, 1.3 Hz, 1H), 4.42 (q, *J* = 7.2 Hz, 2H), 1.37 (t, *J* = 7.2 Hz, 3H). ^13^C{^1^H} NMR (176 MHz, D_2_O) *δ* 164.6, 163.0 (q, *J* = 35.5 Hz), 156.2, 147.7, 137.8, 134.2, 127.6, 122.5, 116.3 (q, *J* = 291.7 Hz), 96.3, 63.2, 13.4. ^19^F NMR (565 MHz, D_2_O) *δ* −75.6. MS (ESI^+^), found: 270.9976 [M − TFA]^+^; calcd. for C_10_H_11_N_2_O_2_ (^80^Se): 270.9981.

Compound **2c** was prepared according to Method A. 2-Pyridylselenyl chloride (50 mg, 0.26 mmol) and 2-cyanoacetamide (22 mg, 0.26 mmol) were used. Yield: 63 mg (81%). ^1^H NMR (700 MHz, D_2_O) *δ* 9.14 (d, *J* = 6.9 Hz, 1H), 8.60 (d, *J* = 8.7 Hz, 1H), 8.24 (t, *J* = 8.5, 1H), 7.98 (td, *J* = 7.1, 1.2 Hz, 1H). ^13^C{^1^H} NMR (176 MHz, D_2_O) *δ* 166.6, 154.8, 147.0, 137.7, 134.0, 127.3, 122.6, 99.1. ^77^Se NMR (95 MHz, MeOH-*d*_4_) *δ* 526.2. MS (ESI^+^), found: 241.9822 [M − Cl]^+^; calcd. for C_8_H_8_N_3_OSe (^80^Se): 241.9827.

Compound **2d** was prepared according to Method B. 2,2′-Py_2_Se_2_ (25 mg, 0.08 mmol), 2-cyanoacetamide (14 mg, 0.16 mmol), and PIFA (35 mg, 0.08 mmol) were used. Yield: 27 mg (69%). ^1^H NMR (700 MHz, D_2_O) *δ* 9.14 (d, *J* = 6.8 Hz, 1H), 8.60 (d, *J* = 8.6 Hz, 1H), 8.24 (t, *J* = 7.9 Hz, 1H), 7.97 (t, *J* = 7.0 Hz, 1H). ^13^C{^1^H} NMR (176 MHz, D_2_O) *δ* 166.6, 163.0 (q, *J* = 35.4 Hz), 154.8, 147.0, 137.7, 134.0, 127.3, 122.6, 116.3 (q, *J* = 291.5 Hz), 99.1. MS (ESI^+^), found: 241.9819 [M − TFA]^+^; calcd. for C_8_H_8_N_3_OSe^+^ (^80^Se): 241.9827.

Compound **2e** was prepared according to Method A. 2-Pyridylselenyl chloride (50 mg, 0.26 mmol) and benzoylacetonitrile (39 mg, 0.27 mmol) were used. Yield: 62 mg (71%). ^1^H NMR (600 MHz, D_2_O) *δ* 9.25 (dt, *J* = 7.0, 1.0 Hz, 1H), 8.57 (dt, *J* = 8.7, 1.1 Hz, 1H), 8.26 (t, *J* = 8.6 Hz, 1H), 7.99 (td, *J* = 7.1, 1.3 Hz, 1H), 7.84–7.80 (m, 2H), 7.72–7.68 (m, 1H), 7.61 (tt, *J* = 7.6, 1.6 Hz, 2H). ^13^C{^1^H} NMR (176 MHz, D_2_O) *δ* 192.0, 157.3, 149.3, 139.5, 138.6, 134.4, 133.2, 129.4, 127.5, 127.3, 122.8, 101.4. ^77^Se NMR (95 MHz, MeOH-*d*_4_) *δ* 527.1. MS (ESI^+^), found: 303.0034 [M − Cl]^+^; calcd. for C_14_H_11_N_2_O (^80^Se) 303.0031.

Compound **2f** was prepared according to Method B. 2,2′-Py_2_Se_2_ (50 mg, 0.16 mmol), benzoylacetonitrile (47 mg, 0.32 mmol), and PIFA (70 mg, 0.16 mmol) were used. Yield: 49 mg (83%). ^1^H NMR (600 MHz, D_2_O) *δ* 9.22 (d, *J* = 6.9 Hz, 1H), 8.55 (d, *J* = 8.6 Hz, 1H), 8.26 (t, *J* = 8.6, 1H), 7.98 (td, *J* = 7.1, 1.3 Hz, 1H), 7.78 (dt, *J* = 8.4, 1.6 Hz, 2H), 7.69–7.63 (m, 1H), 7.57 (t, *J* = 7.7 Hz, 2H). ^13^C{^1^H} NMR (176 MHz, D_2_O) *δ* 191.7, 163.0 (d, *J* = 35.5 Hz), 157.2, 149.3, 139.3, 138.7, 134.4, 133.2, 129.3, 127.5, 127.3, 122.9, 116.4 (d, *J* = 291.6 Hz) 101.1. ^19^F NMR (565 MHz, D_2_O) *δ* −75.5. MS (ESI^+^), found: 303.0028 [M − TFA]^+^; calcd. for C_14_H_11_N_2_OSe^+^ (^80^Se): 303.0031.

Compound **2g** was prepared according to Method A. 2-Pyridylselenyl chloride (20 mg, 0.11 mmol) and malononitrile (69 mg, 0.11 mmol) were used. Yield: 24 mg (89%). IR (*ν*): 2203 cm^−1^ (C≡N). ^1^H NMR (700 MHz, D_2_O) *δ* 9.15 (d, *J* = 6.9 Hz, 1H), 8.60 (d, *J* = 8.8, Hz, 1H), 8.30 (dt, *J* = 8.5, 7.3 Hz, 1H), 8.00 (tt, *J* = 7.1, 1.1 Hz, 1H). ^13^C{^1^H} NMR (176 MHz, D_2_O) *δ* 157.6, 151.4, 138.6, 134.5, 127.6, 123.1, 113.6, 75.4. MS (ESI^+^), found: 223.9731 [M − Cl]^+^; calcd. for C_8_H_6_N_3_Se (^80^Se): 223.9722.

Compound **2h** was prepared according to Method B. 2,2′-Py_2_Se_2_ (100 mg, 0.32 mmol), malononitrile (47 mg, 0.70 mmol), and PIFA (137 mg, 0.32 mmol) were used. Yield: 92 mg (43%). ^1^H NMR (600 MHz, D_2_O) *δ* 9.13 (d, *J* = 6.9 Hz, 1H), 8.58 (d, *J* = 9.3 Hz, 1H), 8.28 (t, *J* = 8.0 Hz, 1H), 7.99 (t, *J* = 7.1 Hz, 1H). ^13^C{^1^H} NMR (176 MHz, D_2_O) *δ* 163.0 (d, *J* = 35.2 Hz), 157.5, 151.3, 138.4, 134.3, 127.4, 123.0, 116.4 (d, *J* = 291.6 Hz), 113.4, 75.2. MS (ESI^+^), found: 223.9725 [M − TFA]^+^; calcd. for C_8_H_6_N_3_Se^+^ (^80^Se): 223.9722.

Compound **2i**: 2-pyridylselenyl chloride (74 mg, 0.38 mmol) and **3b** (98 mg, 0.38 mmol) were stirred in methanol for 30 h. Formed yellow precipitate was separated, washed with an Et_2_O/MeOH 1:1 mixture (1 mL), then with Et_2_O (1 mL) and dried in vacuo. Yield: 144 mg (84%). ^1^H NMR (700 MHz, D_2_O) δ 9.27 (dd, *J* = 9.5, 6.9 Hz, 4H), 8.97 (d, *J* = 8.7 Hz, 2H), 8.75 (d, *J* = 8.7 Hz, 2H), 8.51 (t, *J* = 8.0 Hz, 2H), 8.36 (t, *J* = 8.0 Hz, 2H), 8.08 (dt, *J* = 10.6, 7.0 Hz, 4H). ^13^C{^1^H} NMR (176 MHz, D_2_O) *δ* 168.1, 157.4, 148.6, 145.0, 140.3, 137.8, 136.5, 134.5, 127.4, 126.5, 123.8, 123.0, 93.38. MS (ESI^+^), found: 416.8936 [M − Cl]^+^; calcd. for C_13_H_10_ClN_4_Se_2_^+^ (^80^Se) 416.8919.

Compound **3a** was prepared according to Method C. 2-Pyridylselenyl chloride (30 mg, 0.16 mmol) and ethylcyanoacetate (166 µL, 1.56 mmol) were used. Yield: 35 mg (74%). ^1^H NMR (700 MHz, MeOH-*d*_4_) *δ* 9.58 (d, *J* = 6.7 Hz, 1H), 9.04 (d, *J* = 8.7 Hz, 1H), 8.46 (t, *J* = 7.9 Hz, 1H), 8.06 (t, *J* = 7.0 Hz, 1H), 4.66 (s, 2H, CH_2_), 4.26 (q, *J* = 7.1 Hz, 2H, CH_2_CH_3_), 1.29 (t, *J* = 7.1 Hz, 3H, CH_3_). ^13^C{^1^H} NMR (176 MHz, MeOH-*d*_4_) *δ* 170.4, 168.1, 153.5, 140.34, 138.3, 127.8, 124.0, 63.5, 38.3, 14.3. ^77^Se NMR (95 MHz, MeOH-*d*_4_) *δ* 1017.1. MS (ESI^+^), found: 270.9985 [M − Cl]^+^; calcd. for C_10_H_11_N_2_O_2_Se^+^ (^80^Se) 270.9981.

Compound **3b** was prepared according to Method C. 2-Pyridylselenyl chloride (50 mg, 0.26 mmol) and malononitrile (24 mg, 0.36 mmol) were used. Yield 41 mg (61%). IR (*ν*): 2266 cm^−1^ (C≡N). ^1^H NMR (700 MHz, D_2_O) *δ* 9.35 (d, *J* = 6.6 Hz, 1H), 8.89 (d, *J* = 8.5 Hz, 1H), 8.47 (tt, *J* = 8.3, 1.1 Hz, 1H), 8.10 (td, *J* = 7.1, 1.3 Hz, 1H). ^13^C{^1^H} NMR (176 MHz, D_2_O) *δ* 168.7, 148.4, 139.9, 135.5, 126.3, 123.5, 113.6. ^1^H NMR (700 MHz, MeOH-*d*_4_) *δ* 9.58 (d, *J* = 6.7 Hz, 1H), 9.04 (d, *J* = 8.6 Hz, 1H), 8.46 (t, *J* = 7.9 Hz, 1H), 8.05 (t, *J* = 6.9 Hz, 1H), 4.66 (s, 2H). ^13^C{^1^H} NMR (176 MHz, MeOH-*d*_4_) *δ* 170.4, 168.1, 153.5, 140.4, 138.3, 127.8, 124.0, 38.3. ^77^Se NMR (95 MHz, MeOH-*d*_4_) *δ* 1029.7. MS (ESI^+^), found: 223.9733 [M − Cl]^+^; calcd. for C_8_H_6_N_3_Se^+^ (^80^Se): 223.9722.

Compound **3c**: 2-pyridylselenyl chloride (20 mg, 0.10 mmol) and 1-(cyanomethyl)pyridinium chloride (12 mg, 0.10 mmol) were stirred in MeOH (3 mL) for 6 h. The addition of Et_2_O (1 mL) resulted in the formation of yellow precipitate, which was filtered and washed with Et_2_O/MeOH (1:1). Yield: 20 mg (70%). ^1^H NMR (600 MHz, D_2_O) *δ* 9.52 (d, *J* = 6.7 Hz, 1H), 9.06 (d, *J* = 5.7 Hz, 1H), 8.93 (d, *J* = 8.7 Hz, 1H), 8.81 (t, *J* = 7.9 Hz, 1H), 8.53 (t, *J* = 8.0 Hz, 1H), 8.29 (d, *J* = 11.3 Hz, 2H), 8.19 (t, *J* = 7.0 Hz, 2H), 6.70 (d, *J* = 10.2 Hz, 1H). ^13^C{^1^H} NMR (176 MHz, D_2_O) *δ* 169.1, 148.1, 146.3, 140.4, 135.2, 128.7, 126.6, 123.8, 59.9. MS (ESI^+^), found: 276.0042 [M − 2Cl − H]^+^; calcd. for C_12_H_10_N_3_Se^2+^ (^80^Se): 276.0034.

Compound **3d**: 2-pyridylselenyl chloride (42.2 mg, 0.22 mmol) and N-(cyanomethyl)-4-dimethylaminopyridinium chloride (43.3 mg, 0.22 mmol) were stirred in MeOH (3 mL) for 6 h. The product was crystallized by slow addition of Et_2_O. Formed yellow crystals were filtered and washed with an Et_2_O/MeOH 1:1 mixture. Yield: 73.5 mg (86%). ^1^H NMR (700 MHz, D_2_O) *δ* 9.43 (d, *J* = 6.7 Hz, 1H), 8.91 (d, *J* = 8.7 Hz, 1H), 8.50 (t, *J* = 8.0 Hz, 1H), 8.14 (t, *J* = 7.0 Hz, 1H), 8.10 (d, *J* = 7.6 Hz, 2H), 7.03 (d, *J* = 7.6 Hz, 2H), 6.14 (s, 2H), 3.29 (s, 6H). ^13^C NMR (176 MHz, D_2_O) *δ* 169.0, 156.9, 152.1, 142.4, 140.2, 135.3, 126.5, 123.6, 107.9, 56.3, 39.7. MS (ESI^+^), found: 160.0275 [M − 2Cl]^2+^; calcd. for C_14_H_16_N_4_Se^2+^ (^80^Se): 160.0265.

Substitution reactions

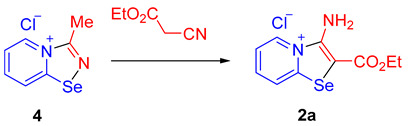

Compounds **2a** and **4** (11.0 mg, 0.047 mmol) and ethylcyanoacetate (50 µL, 0.47 mmol) were dissolved in DCM (0.5 mL) in a stoppered vial at 35 °C, and the mixture was kept for 24 h. After 24 h, formed yellow solid was filtered, washed with Et_2_O (3 mL), and dried in vacuum. Yield: 11.5 mg (86%). Analytical data were in accord with what we had observed previously [2].

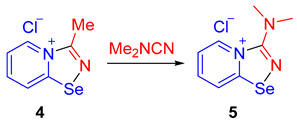

Compounds **5** and **4** (10.2 mg, 0.043 mmol) and dimethycyanamide (30.1 mg, 0.43 mmol) were dissolved in DCM (0.5 mL) in a stoppered vial at 35 °C, and the mixture was kept for 24 h. After 24 h, Et_2_O (0.5 mL) was added, and formed yellow solid was filtered, washed with Et_2_O (3 mL), and dried in vacuum. Yield: 11.2 mg (97%). Analytical data were in accord with what we had observed previously [3].

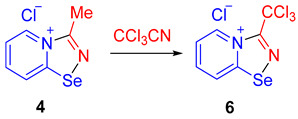

Compounds **6** and **4** (11.1 mg, 0.047 mmol) and trichloroacetonitrile (50 µL, 0.54 mmol) were dissolved in DCM (0.5 mL) in a stoppered vial at 35 °C, and the mixture was kept for 24 h. After 24 h, formed colorless solid was filtered, washed with Et_2_O (3 mL), and dried in vacuum. Yield: 14.9 mg (89%). Analytical data were in accord with what we had observed previously [4].

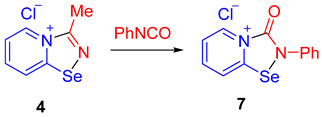

Compounds **7** and **4** (11.1 mg, 0.047 mmol) and phenylisocyanate (7 µL, 0.061 mmol) were dissolved in DCM (0.5 mL) in a stoppered vial at 35 °C, and the mixture was kept for 24 h. After 24 h, formed yellow solid was filtered, washed with Et_2_O (3 mL), and dried in vacuum. Yield: 11.5 mg (86%). Analytical data were in accord with what we had observed previously [2].

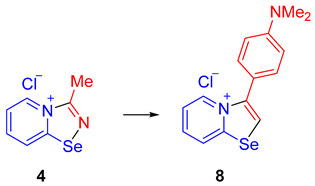

Compounds **8** and **4** (10.6 mg, 0.045 mmol) and 4-dimethylaminophenylacetylene (8 mg, 0.05 mmol) were dissolved in DCM (0.5 mL) in a stoppered vial at RT, and the mixture was kept for 24 h. The formed precipitate was washed with Et_2_O (1.3 mL) and dried in vacuum. Yield: 13.0 mg (85%). ^1^H NMR (600 MHz, D_2_O) *δ* 8.95 (d, *J* = 6.8 Hz, 1H), 8.65 (d, *J* = 8.6 Hz, 1H), 8.14 (ddd, *J* = 8.5, 7.4, 1.0 Hz, 1H), 7.77 (td, *J* = 7.1, 1.1 Hz, 1H), 7.50–7.45 (m, 2H), 7.10–7.04 (m, 2H), 3.00 (s, 6H). ^13^C NMR (151 MHz, D_2_O) *δ* 158.51, 152.78, 142.85, 136.02, 135.64, 131.22, 127.17, 123.68, 122.08, 116.09, 114.09, 40.12.

### 3.2. Theoretical Methods

The geometries and energies of all systems included in the mechanistic study of Figure 1 were fully optimized at the PBE0-D4/def2-TZVP level of theory using the program ORCA version 5.1 [67]. For the calculations, we used the hybrid PBE0 [68] functional with D4 correction for the dispersion [69] and triple-ζ basis set [70]. The minimum and transition state nature of the complexes and compounds have been confirmed by performing frequency calculations. The transition states were located using the Nudged Elastic Band (NEB) tool of ORCA. The NEB method was used to find a minimum energy path (MEP) connecting given reactant and product state minima on the energy surface. An initial path was generated and represented by a discrete set of configurations of the atoms, referred to as images of the system. The implementation in ORCA is described in detail in the article by Ásgeirsson et al. [71].

The single-point calculations using the X-ray coordinates of compounds **2a** (a), **2c** (b,c), **2e** (d), **2g** (e), and **3b** were performed using the Turbomole 7.7 program [72]. The level of theory used was the same used for the mechanistic study PBE0-D4/def2-TZVP. The MEP surfaces were generated at the same level of theory and the 0.001 a.u. isosurface. The QTAIM [73] distribution of CPs and bond paths and NCIplot RDG isosurfaces [74] were computed using the MultiWFN program [75] and plotted using the VMD program [76]. The following settings were used for the RDG plots: s = 0.45 a.u.; cut-off ρ = 0.04 a.u.; and color scale −0.025 a.u. ≤ sign(λ_2_)ρ ≤ 0.025 a.u. The NBO analysis [77] was performed at the same level using the NBO 7.0 program [78].

## 4. Conclusions

Overall, we have studied the coupling between nitriles containing an active α-methylene group and ambiphilic 2-pyridylselenyl reagents. Depending on the conditions employed, two different Se-containing heterocyclic scaffolds can be accessed in high yields. A combination of experimental and theoretical analyses revealed two distinct cyclization pathways leading to either selenadiazolium or selenazolium products. The formation of the first type of heterocycle turned out to be reversible, gradually isomerizing in a solution into the thermodynamically more stable selenazolium product.

Importantly, we demonstrated for the first time that 1,3-dipolar cyclization between the CN triple bond and 2-pyridylselenyl reagents is reversible. This reversibility was confirmed through various substitution experiments, showcasing the dynamic nature of the 1,3-dipolar cyclization. This finding is promising from the perspective of dynamic covalent chemistry, and the research into applications of 1,3-dipolar cyclization between nitriles and 2-pyridylselenyl reagents in supramolecular chemistry is currently underway in our laboratory.

## Data Availability

The data presented in this study are available.

## Supplementary Materials

The following supporting information can be downloaded at: https://www.mdpi.com/article/10.3390/ijms252312798/s1. Refs. [79,80,81,82,83,84,85] cited in Supplementary.

## References

[1] Wu X.-F., Neumann H., Beller M. (2013). Synthesis of Heterocycles via Palladium-Catalyzed Carbonylations. Chem. Rev..

[2] Deiters A., Martin S.F. (2004). Synthesis of Oxygen- and Nitrogen-Containing Heterocycles by Ring-Closing Metathesis. Chem. Rev..

[3] Tu L., Gao L., Wang X., Shi R., Ma R., Li J., Lan X., Zheng Y., Liu J. (2021). [3 + 2] Cycloaddition of Nitrile Imines with Enamides: An Approach to Functionalized Pyrazolines and Pyrazoles. J. Org. Chem..

[4] (1986). 1,3-Dipolar Cycloaddition Chemistry. Volumes 1 and 2. Edited by Albert Padwa. John Wiley and Sons. New York, 1984. Volume 1: XIII + 817 Pages. Volume 2: XIII + 704 Pages. ISBN 0–471–08364-X (Set). $295.00 for the Two-Volume Set. J. Heterocycl. Chem..

[5] Bokach N.A., Kukushkin V.Y. (2006). 1,3-Dipolar Cycloaddition of Nitrones to Free and Coordinated Nitriles: Routes to Control the Synthesis of 2,3-Dihydro-1,2,4-Oxadiazoles. Russ. Chem. Bull..

[6] Bokach N.A., Kuznetsov M.L., Kukushkin V.Y. (2011). 1,3-Dipolar Cycloaddition of Nitrone-Type Dipoles to Uncomplexed and Metal-Bound Substrates Bearing the CN Triple Bond. Coord. Chem. Rev..

[7] Hermkens P.H.H., Maarseveen J.H., Kruse C.G., Scheeren H.W. (1988). 1,3-Dipolar Cycloaddition of Nitrones with Nitriles.: Scope and Mechanistic Study. Tetrahedron.

[8] Troisi L., Ronzini L., Rosato F., Videtta V. (2009). [3 + 2] Cycloaddition of Oxaziridines with Nitriles: Synthesis of 2,3-Dihydro-1,2,4-Oxadiazoles. Synlett.

[9] Nascimento M.A., LaPierre E.A., Patrick B.O., Watson J.E.T., Watanabe L., Rawson J., Hering-Junghans C., Manners I. (2024). 1,3-Dipolar Cyclisation Reactions of Nitriles with Sterically Encumbered Cyclic Triphosphanes: Synthesis and Electronic Structure of Phosphorus-Rich Heterocycles with Tunable Colour. Chem. Sci..

[10] Fairlie D.P., Jackson W.G., Skelton B.W., Wen H., White A.H., Wickramasinghe W.A., Woon T.C., Taube H. (1997). Models for Arginine−Metal Binding. Synthesis of Guanidine and Urea Ligands through Amination and Hydration of a Cyanamide Ligand Bound to Platinum(II), Osmium(III), and Cobalt(III). Inorg. Chem..

[11] Kukushkin V.Y., Pombeiro A.J.L. (2002). Additions to Metal-Activated Organonitriles. Chem. Rev..

[12] Michelin R.A., Mozzon M., Bertani R. (1996). Reactions of Transition Metal-Coordinated Nitriles. Coord. Chem. Rev..

[13] Astafiev A.A., Shakhov A.M., Kritchenkov A.S., Khrustalev V.N., Shepel D.V., Nadtochenko V.A., Tskhovrebov A.G. (2021). Femtosecond Laser Synthesis of Nitrogen-Doped Luminescent Carbon Dots from Acetonitrile. Dyes Pigments.

[14] Khrustalev V.N., Grishina M.M., Matsulevich Z.V., Lukiyanova J.M., Borisova G.N., Osmanov V.K., Novikov A.S., Kirichuk A.A., Borisov A.V., Solari E. (2021). Novel Cationic 1,2,4-Selenadiazoles: Synthesis via Addition of 2-Pyridylselenyl Halides to Unactivated Nitriles, Structures and Four-Center Se⋯N Contacts. Dalton Trans..

[15] Grudova M.V., Khrustalev V.N., Kubasov A.S., Strashnov P.V., Matsulevich Z.V., Lukiyanova J.M., Borisova G.N., Kritchenkov A.S., Grishina M.M., Artemjev A.A. (2022). Adducts of 2-Pyridylselenenyl Halides and Nitriles as Novel Supramolecular Building Blocks: Four-Center Se···N Chalcogen Bonding versus Other Weak Interactions. Cryst. Growth Des..

[16] Artemjev A.A., Novikov A.P., Burkin G.M., Sapronov A.A., Kubasov A.S., Nenajdenko V.G., Khrustalev V.N., Borisov A.V., Kirichuk A.A., Kritchenkov A.S. (2022). Towards Anion Recognition and Precipitation with Water-Soluble 1,2,4-Selenodiazolium Salts: Combined Structural and Theoretical Study. Int. J. Mol. Sci..

[17] Grudova M.V., Kubasov A.S., Khrustalev V.N., Novikov A.S., Kritchenkov A.S., Nenajdenko V.G., Borisov A.V., Tskhovrebov A.G. (2022). Exploring Supramolecular Assembly Space of Cationic 1,2,4-Selenodiazoles: Effect of the Substituent at the Carbon Atom and Anions. Molecules.

[18] Tskhovrebov A.G., Luzyanin K.V., Dolgushin F.M., Guedes Da Silva M.F.C., Pombeiro A.J.L., Kukushkin V.Y. (2011). Novel Reactivity Mode of Metal Diaminocarbenes: Palladium(II)-Mediated Coupling between Acyclic Diaminocarbenes and Isonitriles Leading to Dinuclear Species. Organometallics.

[19] Kazakova A.A., Kubasov A.S., Chizhov A.O., Novikov A.P., Volkov M.A., Borisov A.V., Nenajdenko V.G., Dukhnovsky E.A., Bely A.E., Grishina M.M. (2024). Perrhenate and Pertechnetate Complexes of Dicationic Pyridinium-Fused 1,2,4-Selenodiazoles Featuring Se⋯O Chalcogen Bonding and Anion⋯anion Interactions. Inorganica Chim. Acta.

[20] Buslov I.V., Novikov A.S., Khrustalev V.N., Grudova M.V., Kubasov A.S., Matsulevich Z.V., Borisov A.V., Lukiyanova J.M., Grishina M.M., Kirichuk A.A. (2021). 2-Pyridylselenenyl versus 2-Pyridyltellurenyl Halides: Symmetrical Chalcogen Bonding in the Solid State and Reactivity towards Nitriles. Symmetry.

[21] Sapronov A.A., Artemjev A.A., Burkin G.M., Khrustalev V.N., Kubasov A.S., Nenajdenko V.G., Gomila R.M., Frontera A., Kritchenkov A.S., Tskhovrebov A.G. (2022). Robust Supramolecular Dimers Derived from Benzylic-Substituted 1,2,4-Selenodiazolium Salts Featuring Selenium⋯π Chalcogen Bonding. Int. J. Mol. Sci..

[22] Artemjev A.A., Kubasov A.S., Kuznetsov M.L., Grudova M.V., Khrustalev V.N., Kritchenkov A.S., Tskhovrebov A.G. (2023). Mechanistic Investigation of 1,3-Dipolar Cycloaddition between Bifunctional 2-Pyridylselenyl Reagents and Nitriles Including Reactions with Cyanamides. CrystEngComm.

[23] Sapronov A.A., Khrustalev V.N., Chusova O.G., Kubasov A.S., Kritchenkov A.S., Nenajdenko V.G., Gomila R.M., Frontera A., Tskhovrebov A.G. (2024). Introducing Cationic Selenium-Containing Triazapentadiene Ligand Framework: Synthesis, Coordination Chemistry, and Antifungal Activity. Inorg. Chem..

[24] Power P.P. (2010). Main-Group Elements as Transition Metals. Nature.

[25] Ninomiya M., Garud D.R., Koketsu M. (2011). Biologically Significant Selenium-Containing Heterocycles. Coord. Chem. Rev..

[26] Nogueira C.W., Rocha J.B.T. (2011). Toxicology and Pharmacology of Selenium: Emphasis on Synthetic Organoselenium Compounds. Arch. Toxicol..

[27] Ji S., Xia J., Xu H. (2016). Dynamic Chemistry of Selenium: Se–N and Se–Se Dynamic Covalent Bonds in Polymeric Systems. ACS Macro Lett..

[28] Yi Y., Fa S., Cao W., Zeng L., Wang M., Xu H., Zhang X. (2012). Fabrication of Well-Defined Crystalline Azacalixarene Nanosheets Assisted by Se⋯N Non-Covalent Interactions. Chem. Commun..

[29] Ji S., Cao W., Yu Y., Xu H. (2014). Dynamic Diselenide Bonds: Exchange Reaction Induced by Visible Light without Catalysis. Angew. Chem. Int. Ed..

[30] Artemjev A.A., Kubasov A.S., Zaytsev V.P., Borisov A.V., Kritchenkov A.S., Nenajdenko V.G., Gomila R.M., Frontera A., Tskhovrebov A.G. (2023). Novel Chalcogen Bond Donors Derived from [3 + 2] Cycloaddition Reaction between 2-Pyridylselenyl Reagents and Isocyanates: Synthesis, Structures and Theoretical Studies. Cryst. Growth Des..

[31] Alajarin M., Marin-Luna M., Vidal A. (2012). Recent Highlights in Ketenimine Chemistry. Eur. J. Org. Chem..

[32] Allen F.H., Kennard O., Watson D.G., Brammer L., Orpen A.G., Taylor R. (1987). Tables of Bond Lengths Determined by X-Ray and Neutron Diffraction. Part 1. Bond Lengths in Organic Compounds. J. Chem. Soc. Perkin Trans. 2.

[33] Sapronov A.A., Kubasov A.S., Khrustalev V.N., Artemjev A.A., Burkin G.M., Dukhnovsky E.A., Chizhov A.O., Kritchenkov A.S., Gomila R.M., Frontera A. (2023). Se-Pi Chalcogen Bonding in 1,2,4-Selenodiazolium Tetraphenylborate Complexes. Symmetry.

[34] Dukhnovsky E.A., Novikov A.S., Kubasov A.S., Borisov A.V., Sikaona N.D., Kirichuk A.A., Khrustalev V.N., Kritchenkov A.S., Tskhovrebov A.G. (2024). Halogen Bond-Assisted Supramolecular Dimerization of Pyridinium-Fused 1,2,4-Selenadiazoles via Four-Center Se_2_N_2_ Chalcogen Bonding. Int. J. Mol. Sci..

[35] Tskhovrebov A.G., Solari E., Scopelliti R., Severin K. (2013). Insertion of Zerovalent Nickel into the N-N Bond of N-Heterocyclic-Carbene- Activated N_2_O. Inorg. Chem..

[36] Tskhovrebov A.G., Novikov A.S., Tupertsev B.S., Nazarov A.A., Antonets A.A., Astafiev A.A., Kritchenkov A.S., Kubasov A.S., Nenajdenko V.G., Khrustalev V.N. (2021). Azoimidazole Gold(III) Complexes: Synthesis, Structural Characterization and Self-Assembly in the Solid State. Inorganica Chim. Acta.

[37] Aakeroy C.B., Bryce D.L., Desiraju G.R., Frontera A., Legon A.C., Nicotra F., Rissanen K., Scheiner S., Terraneo G., Metrangolo P. (2019). Definition of the Chalcogen Bond (IUPAC Recommendations 2019). Pure Appl. Chem..

[38] Scilabra P., Terraneo G., Resnati G. (2019). The Chalcogen Bond in Crystalline Solids: A World Parallel to Halogen Bond. Acc. Chem. Res..

[39] Murray J.S., Lane P., Politzer P. (2009). Expansion of the σ-Hole Concept. J. Mol. Model..

[40] Benz S., López-Andarias J., Mareda J., Sakai N., Matile S. (2017). Catalysis with Chalcogen Bonds. Angew. Chem. Int. Ed..

[41] Mahmudov K.T., Kopylovich M.N., Guedes da Silva M.F.C., Pombeiro A.J.L. (2017). Chalcogen Bonding in Synthesis, Catalysis and Design of Materials. Dalton Trans..

[42] Fick R.J., Kroner G.M., Nepal B., Magnani R., Horowitz S., Houtz R.L., Scheiner S., Trievel R.C. (2016). Sulfur–Oxygen Chalcogen Bonding Mediates AdoMet Recognition in the Lysine Methyltransferase SET7/9. ACS Chem. Biol..

[43] Beno B.R., Yeung K.-S., Bartberger M.D., Pennington L.D., Meanwell N.A. (2015). A Survey of the Role of Noncovalent Sulfur Interactions in Drug Design. J. Med. Chem..

[44] Lim J.Y.C., Marques I., Thompson A.L., Christensen K.E., Félix V., Beer P.D. (2017). Chalcogen Bonding Macrocycles and [2]Rotaxanes for Anion Recognition. J. Am. Chem. Soc..

[45] Bauzá A., Frontera A. (2020). Halogen and Chalcogen Bond Energies Evaluated Using Electron Density Properties. ChemPhysChem.

[46] Espinosa E., Molins E., Lecomte C. (1998). Hydrogen Bond Strengths Revealed by Topological Analyses of Experimentally Observed Electron Densities. Chem. Phys. Lett..

[47] Scheiner S. (2021). Dissection of the Origin of π-Holes and the Noncovalent Bonds in Which They Engage. J. Phys. Chem. A.

[48] Zhao W., Flood A.H., White N.G. (2020). Recognition and applications of anion–anion dimers based on anti-electrostatic hydrogen bonds (AEHBs). Chem. Soc. Rev..

[49] White N.G. (2019). Antielectrostatically hydrogen bonded anion dimers: Counter-intuitive, common and consistent. CrystEngComm.

[50] Zierkiewicz W., Michalczyk M., Maris T., Wysokiński R., Scheiner S. (2021). Experimental and theoretical evidence of attractive interactions between dianions: [PdCl4]2−⋯[PdCl4]2−. Chem. Commun..

[51] Brinck T., Murray J.S., Politzer P. (1992). Surface electrostatic potentials of halogenated methanes as indicators of directional intermolecular interactions. Int. J. Quantum Chem..

[52] Ramasubbu N., Parthasarathy R., Murray-Rust P. (1986). Angular preferences of intermolecular forces around halogen centers: Preferred directions of approach of electrophiles and nucleophiles around carbon-halogen bond. J. Am. Chem. Soc..

[53] Metrangolo P., Resnati G. (2001). Halogen Bonding: A Paradigm in Supramolecular Chemistry. Chem.—A Eur. J..

[54] Lommerse J.P.M., Stone A.J., Taylor R., Allen F.H. (1996). The Nature and Geometry of Intermolecular Interactions between Halogens and Oxygen or Nitrogen. J. Am. Chem. Soc..

[55] Weinhold F. (2022). Anti-Electrostatic Pi-Hole Bonding: How Covalency Conquers Coulombics. Molecules.

[56] Guha A.K. (2023). Can Dative Bond Between Two Anions Possible?. ChemPhysChem.

[57] Tarannam N., Shukla R., Kozuch S. (2021). Yet another perspective on hole interactions. Phys. Chem. Chem. Phys..

[58] Chen L., Feng Q., Wang C., Yin S., Mo Y. (2021). Classical Electrostatics Remains the Driving Force for Interanion Hydrogen and Halogen Bonding. J. Phys. Chem. A.

[59] Wysokiński R., Zierkiewicz W., Michalczyk M., Maris T., Scheiner S. (2022). The Role of Hydrogen Bonds in Interactions between [PdCl_4_]^2−^ Dianions in Crystal. Molecules.

[60] Li Y., Meng L., Zeng Y. (2021). Comparison of Anion-Anion Halogen Bonds with Neutral-Anion Halogen Bonds in the Gas Phase and Polar Solvents. Chempluschem.

[61] Holthoff J.M., Engelage E., Weiss R., Huber S.M. (2020). “Anti-Electrostatic” Halogen Bonding. Angew. Chemie Int. Ed..

[62] Zierkiewicz W., Wysokiński R., Michalczyk M., Scheiner S. (2020). On the Stability of Interactions between Pairs of Anions—Complexes of MCl_3_^−^ (M=Be, Mg, Ca, Sr, Ba) with Pyridine and CN^−^. ChemPhysChem.

[63] Loy C., Holthoff J.M., Weiss R., Huber S.M., Rosokha S. (2021). V “Anti-electrostatic” halogen bonding in solution. Chem. Sci..

[64] Grabowski S.J. (2014). Boron and other Triel Lewis Acid Centers: From Hypovalency to Hypervalency. ChemPhysChem.

[65] Leitz D., Bayer M.C., Morgenstern Y., Zischka F., Kornath A.J. (2018). Tuning the Anomeric Effect in Sulfamide with Superacids. Chem.—A Eur. J..

[66] Nievergelt P.P., Babor M., Čejka J., Spingler B. (2018). A high throughput screening method for the nano-crystallization of salts of organic cations. Chem. Sci..

[67] Neese F. (2022). Software update: The ORCA program system—Version 5.0. WIREs Comput. Mol. Sci..

[68] Adamo C., Barone V. (1999). Toward Reliable Density Functional Methods without Adjustable Parameters: The PBE0 Model. J. Chem. Phys..

[69] Caldeweyher E., Ehlert S., Hansen A., Neugebauer H., Spicher S., Bannwarth C., Grimme S. (2019). A Generally Applicable Atomic-Charge Dependent London Dispersion Correction. J. Chem. Phys..

[70] Weigend F. (2006). Accurate Coulomb-Fitting Basis Sets for H to Rn. Phys. Chem. Chem. Phys..

[71] Ásgeirsson V., Birgirsson B.O., Bjornsson R., Becker U., Riplinger C., Neese F., Jónssson H. (2021). Nudged elastic band method for molecular reactions using energy-weighted springs combined with eigenvector following. J. Chem. Theory Comput..

[72] Ahlrichs R., Bär M., Häser M., Horn H., Kölmel C. (1989). Electronic Structure Calculations on Workstation Computers: The Program System Turbomole. Chem. Phys. Lett..

[73] Bader R.F.W. (1991). A Quantum Theory of Molecular Structure and Its Applications. Chem. Rev..

[74] Contreras-García J., Johnson E.R., Keinan S., Chaudret R., Piquemal J.-P., Beratan D.N., Yang W. (2011). NCIPLOT: A program for plotting non-covalent interaction regions. J. Chem. Theory Comput..

[75] Lu T., Chen F. (2012). Multiwfn: A Multifunctional Wavefunction Analyzer. J. Comput. Chem..

[76] Humphrey W., Dalke A., Schulten K. (1996). VMD: Visual Molecular Dynamics. J. Mol. Graph..

[77] Glendening E.D., Landis C.R., Weinhold F. (2012). Natural Bond Orbital Methods. WIREs Comput. Mol. Sci..

[78] Glendening E.D., Badenhoop J.K., Reed A.E., Carpenter J.E., Bohmann J.A., Morales C.M., Karafiloglou P., Landis C.R., Weinhold F. (2018). NBO 7.0.

[79] Bruker (2019). SAINT.

[80] Krause L., Herbst-Irmer R., Sheldrick G., Stalke D. (2015). Comparison of silver and molybdenum microfocus X-ray sources for single-crystal structure determination. J. Appl. Cryst..

[81] Rigaku (2021). CrysAlisPro Software System.

[82] Sheldrick G.M. (2015). Crystal Structure Refinement with SHELXL. Acta Cryst..

[83] Caldeweyher E., Mewes J.-M., Ehlert S., Grimme S. (2020). Extension and evaluation of the D4 London-dispersion model for periodic systems. Phys. Chem. Chem. Phys..

[84] Bartashevich E.V., Tsirelson V.G. (2014). Interplay between non-covalent interactions in complexes and crystals with halogen bonds. Russ. Chem. Rev..

[85] Glendening E.D., Landis C.R., Weinhold F. (2019). NBO 7.0: New vistas in localized and delocalized chemical bonding theory. J. Comput. Chem..

