# Supplementary material for: Diverse Cyclization Pathways Between Nitriles with Active α-Methylene Group and Ambiphilic 2-Pyridylselenyl Reagents Enabled by Reversible Covalent Bonding
Source: Int J Mol Sci. 2024 Nov 28;25(23):12798. doi: 10.3390/ijms252312798 (PMC11641742; doi:10.3390/ijms252312798)
Supplement: Supplementary file 1 [file ijms-25-12798-s001.zip › ijms-3327168-supplementary.pdf]

International Journal of *Molecular Sciences*

MDPI

Experimental section

X-ray crystal structure determination

The single-crystal X-ray diffraction data were collected on a three-circle Bruker diffractometer equipped with an APEX-II area-detector (T = 100-150 K, MoK_{α}-radiation, graphite monochromator, ω and φ scan mode) (**2a**, **2c-f**, **3b** and **3d**) and a four-circle Rigaku Synergy S diffractometer equipped with a HyPix6000HE area-detector (T = 100 K, CuK_{α}-radiation, graphite monochromator, shutterless ω scan mode) (**2g-i**). For compounds **2a**, **2c-f**, **3b** and **3d**, the data were indexed and integrated using the *SAINT* program [79] and then scaled and corrected for absorption using the *SADABS* program [80]. For compounds **2g-i**, the data were integrated and corrected for absorption by the *CrysAlisPro* program [81]. For details, see Electronic Supporting Information. The structures were solved by direct methods and refined by a full-matrix least squares technique on F^2 with anisotropic displacement parameters for non-hydrogen atoms. The amino-hydrogen atoms in **2a** and **2c-i** as well as the solvate water (**2a** and **2i**) and methanol (**2c**) hydrogen atoms were localized in the difference-Fourier maps and refined within riding model with fixed isotropic displacement parameters [U_{iso}(H) = 1.2U_{eq}(N) and 1.5U_{eq}(C,O)]. The other hydrogen atoms in all compounds were placed in calculated positions and refined within riding model with fixed isotropic displacement parameters [U_{iso}(H) = 1.5U_{eq}(C) for the CH_3-groups and 1.2U_{eq}(C) for the other groups]. All calculations were carried out using the SHELXTL program suite [82].

Crystallographic data for compounds **2a**, **2c-i**, **3b** and **3d** have been deposited with the Cambridge Crystallographic Data Center, CCDC 2375395-2375404, respectively. Copies of this information may be obtained free of charge from the Director, CCDC, 12 Union Road, Cambridge CHB2 1EZ, UK (Fax: +44 1223 336033; e-mail: deposit@ccdc.cam.ac.uk or www.ccdc.cam.ac.uk).

Theoretical methods

In this study, we calculated the energies and wave-functions of the examined compounds and supramolecular assemblies using the RI-BPE0-D4/def2-TZVP theoretical level [68,70] with X-ray coordinates, employing the Turbomole 7.7 program [72]. We incorporated Grimme's D4 dispersion correction [83], which is particularly adept at accurately evaluating non-covalent interactions. For characterizing non-covalent interactions, we employed Bader's QTAIM method [73]. The QTAIM analysis was conducted at the same theoretical level using the MULTIWFN program [75] and visualized through the VMD software [76]. The formation energies of the hydrogen bonds [46] and chalcogen bonds have been estimated by using the value of the potential energy density at the BCPs by using the equations proposed in the literature [45, 46,84]. The NBO analysis [85] was performed using the NBO 7.0 program.

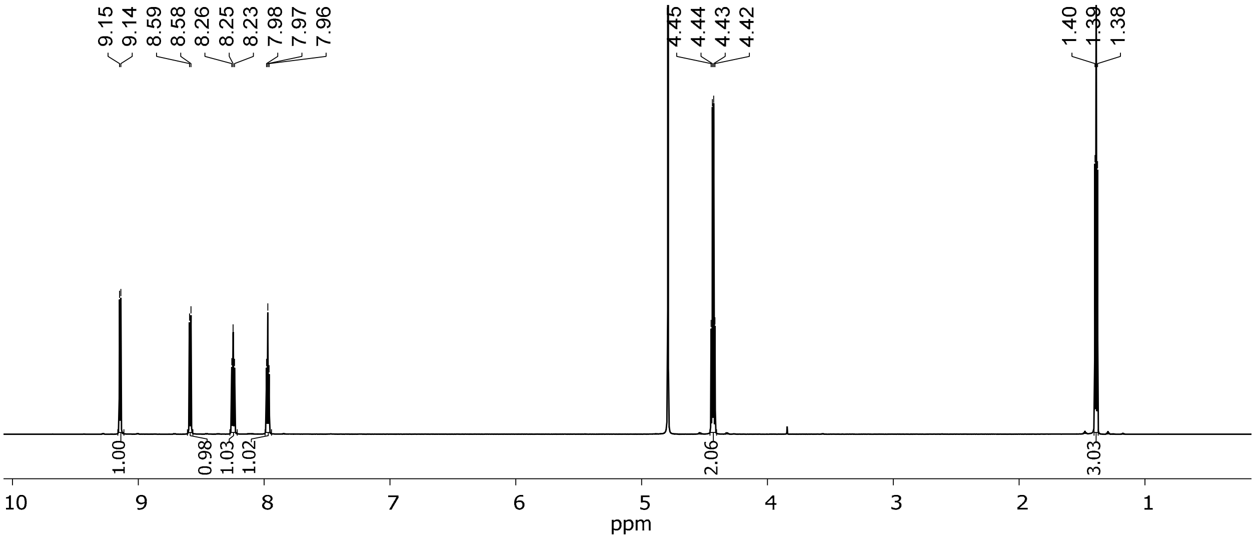

^{1}H NMR spectrum (700 MHz, D_2O) of **2a**

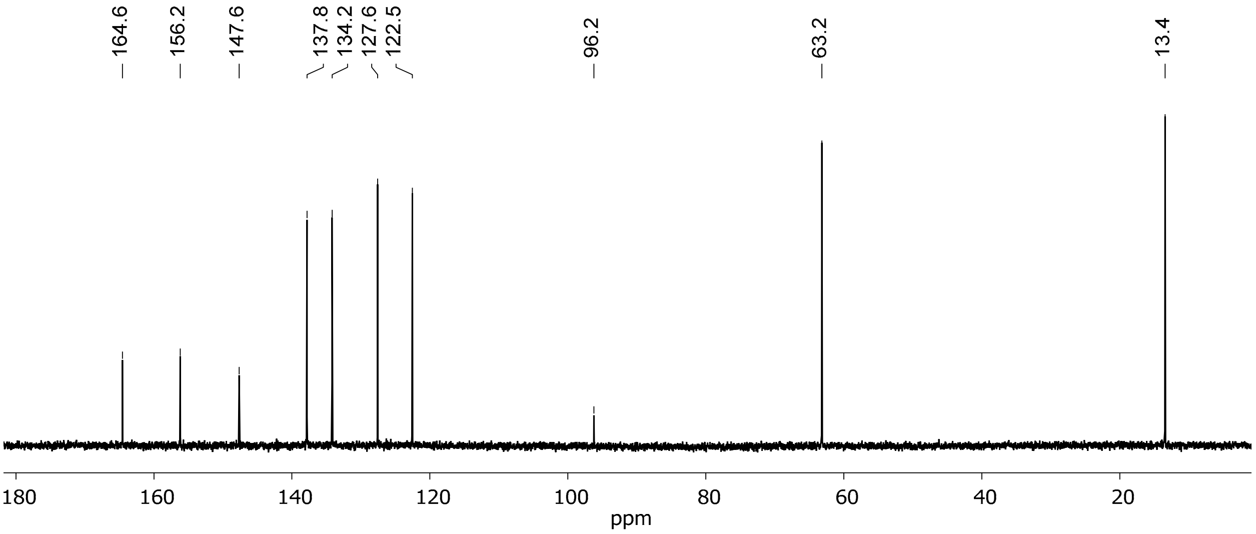

$^{13}C\{H\}$ NMR spectrum (176 MHz, D_2O) of **2a**

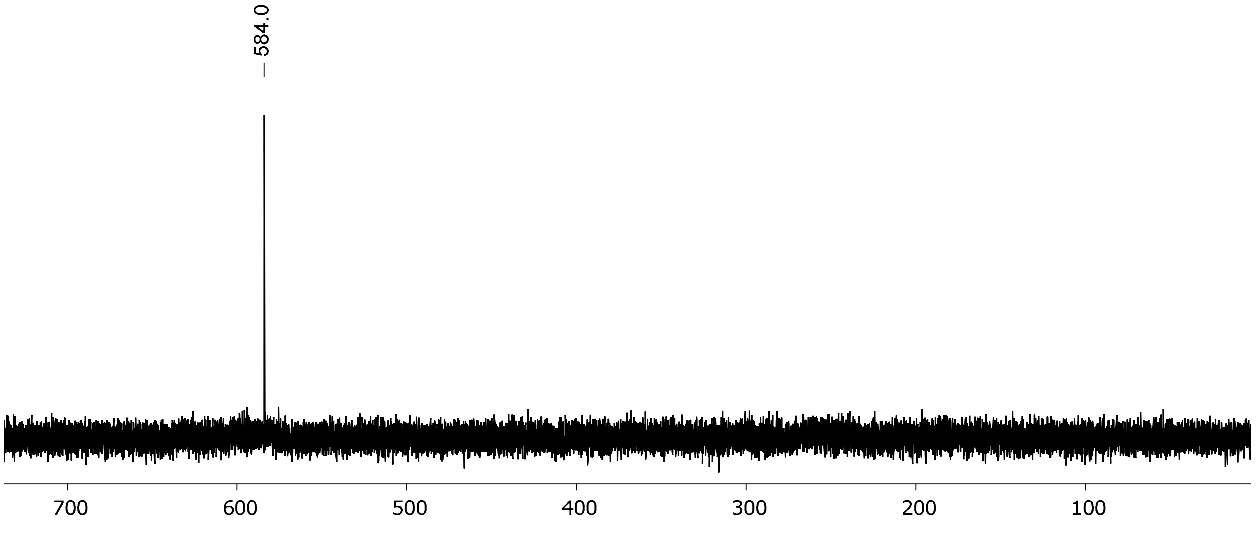

^{77}Se NMR spectrum (95 MHz, MeOH-d_4) of **2a**

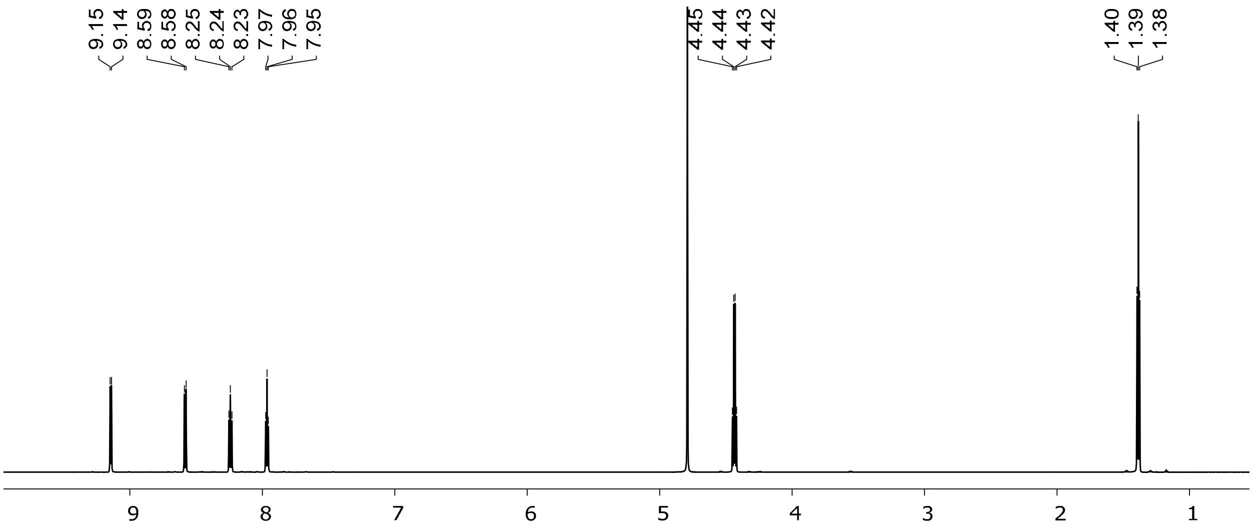

^{1}H NMR spectrum (700 MHz, D_2O) of **2b**

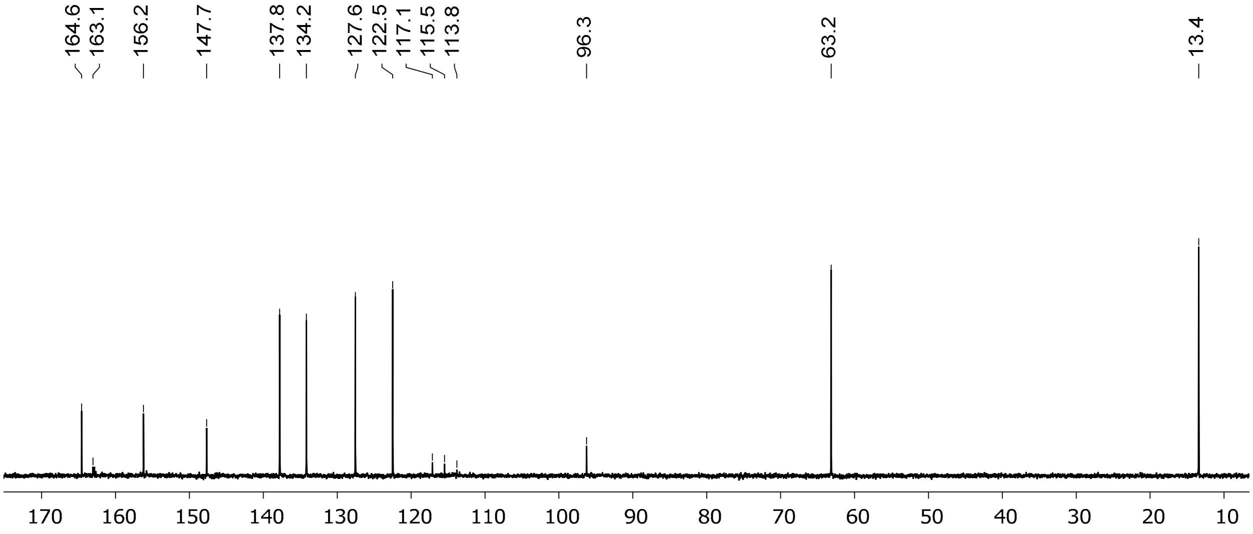

^{13}C NMR spectrum (176 MHz, D_2O) of **2b**

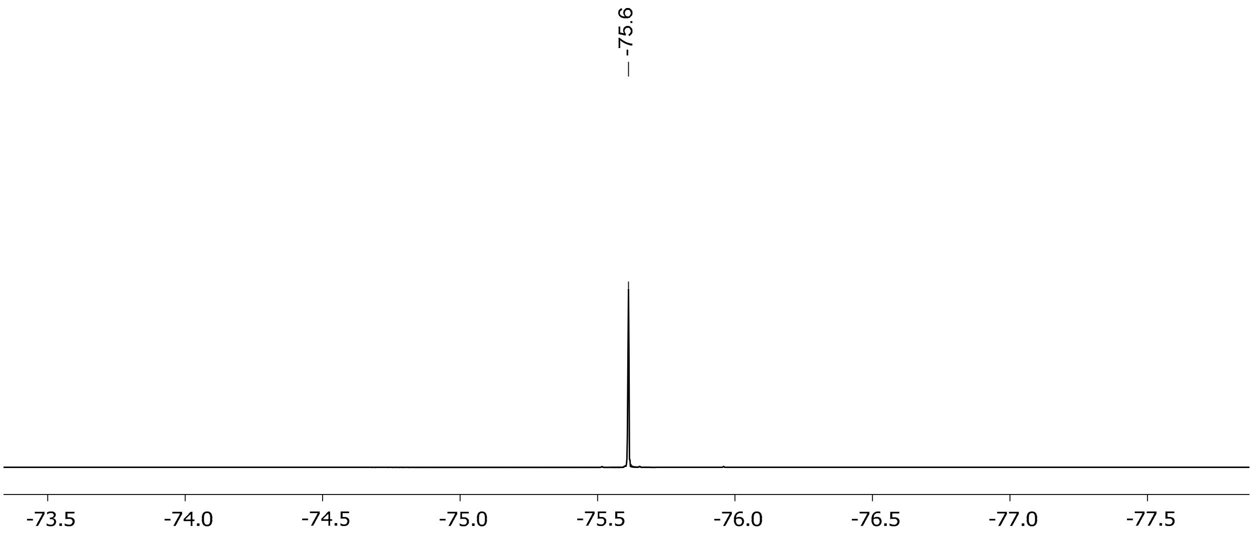

^{19}F NMR spectrum (659 MHz, D_2O) of **2b**

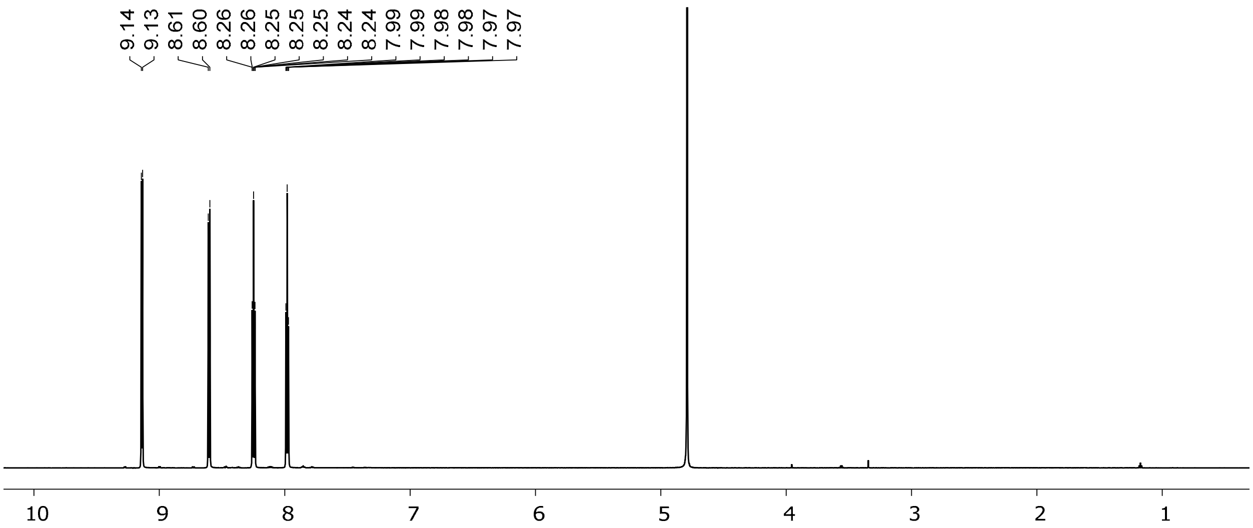

^{1}H NMR spectrum (700 MHz, D_2O) of **2c**

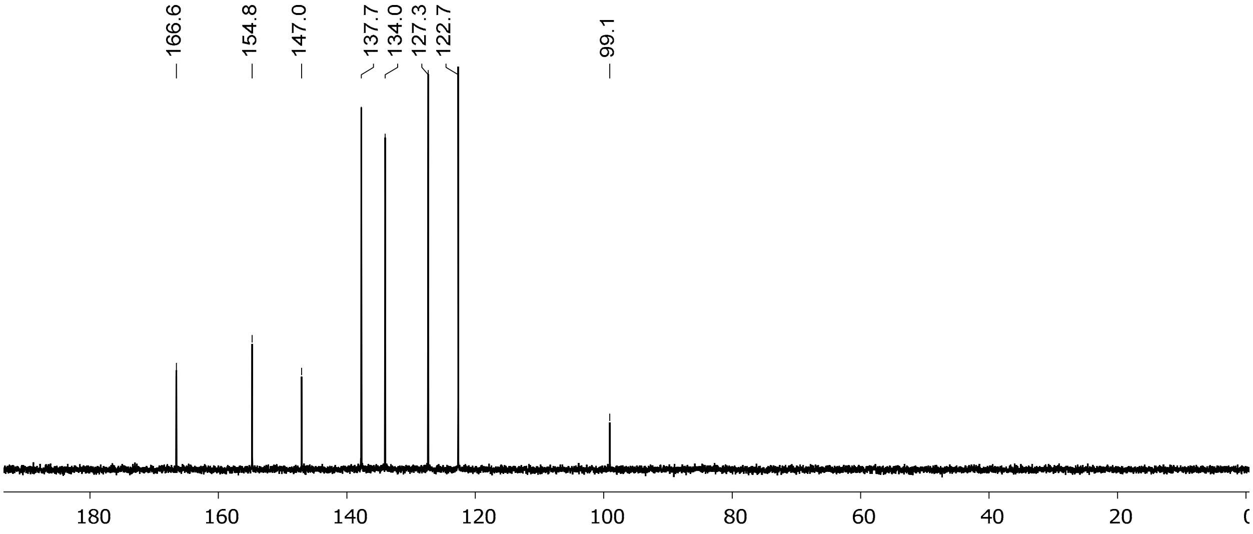

^{13}C NMR spectrum (176 MHz, D_2O) of **2c**

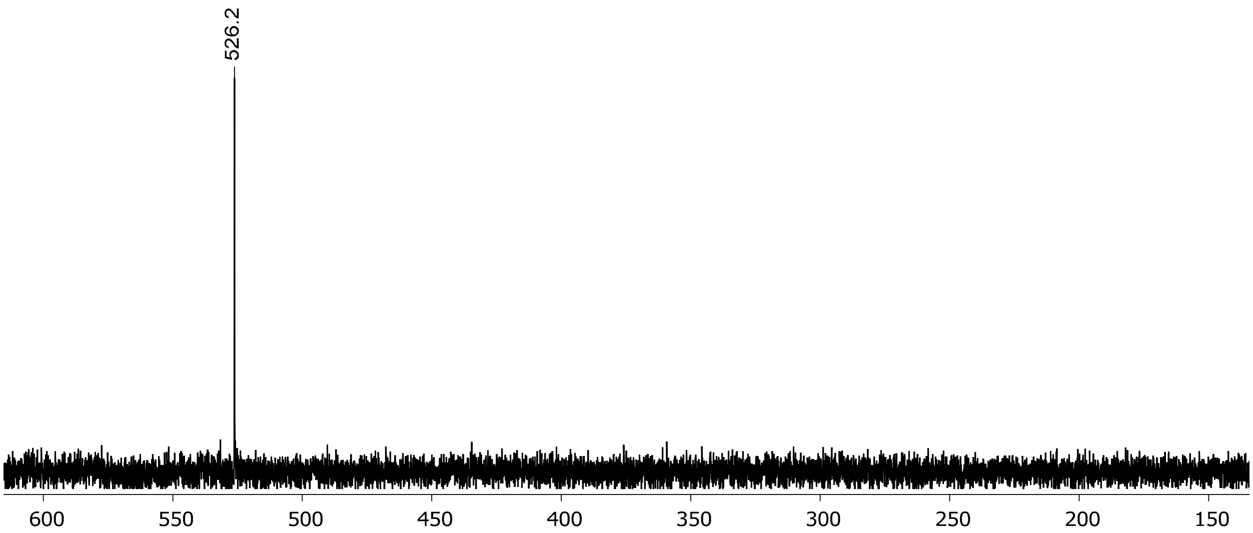

^{77}Se NMR spectrum (95 MHz, MeOH-d_4) of **2c**

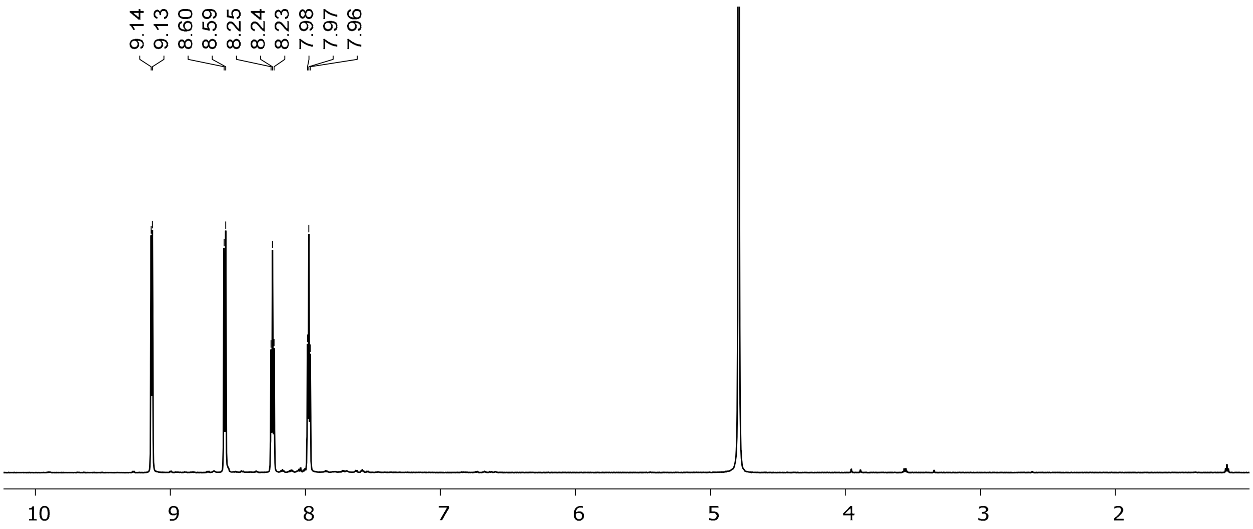

^{1}H NMR spectrum (700 MHz, D_2O) of **2d**

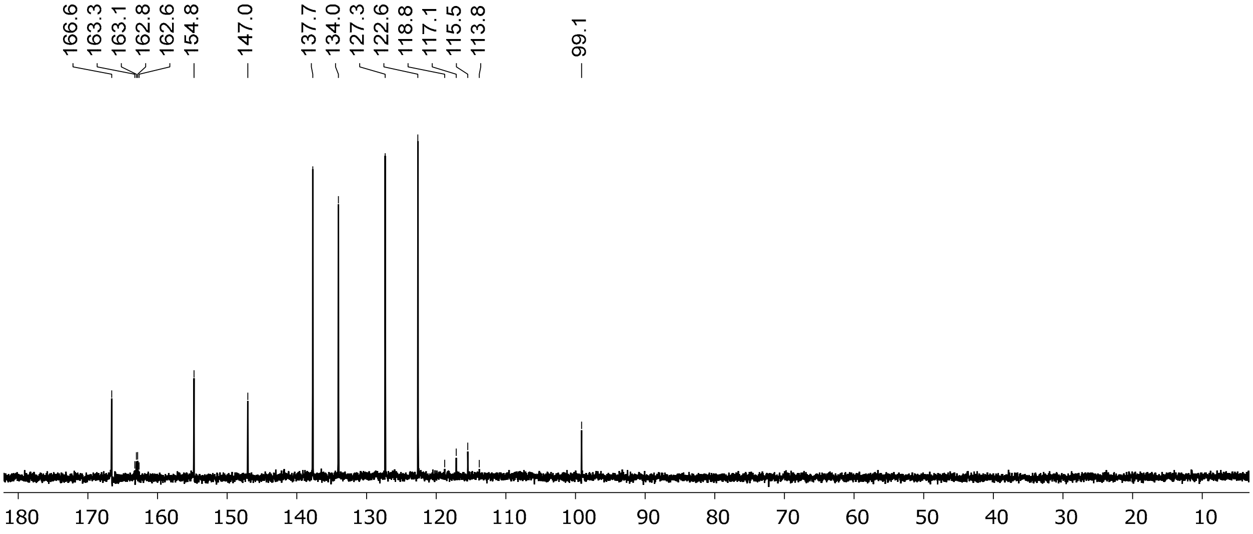

^{13}C NMR spectrum (176 MHz, D_2O) of **2d**

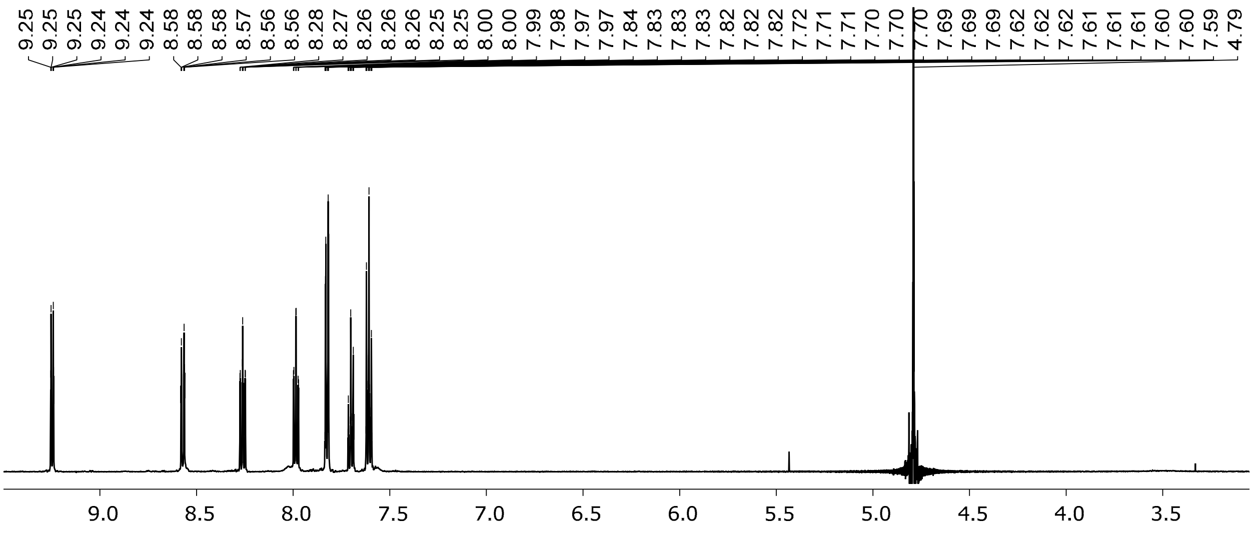

^{1}H NMR spectrum (600 MHz, D_2O) of **2e**

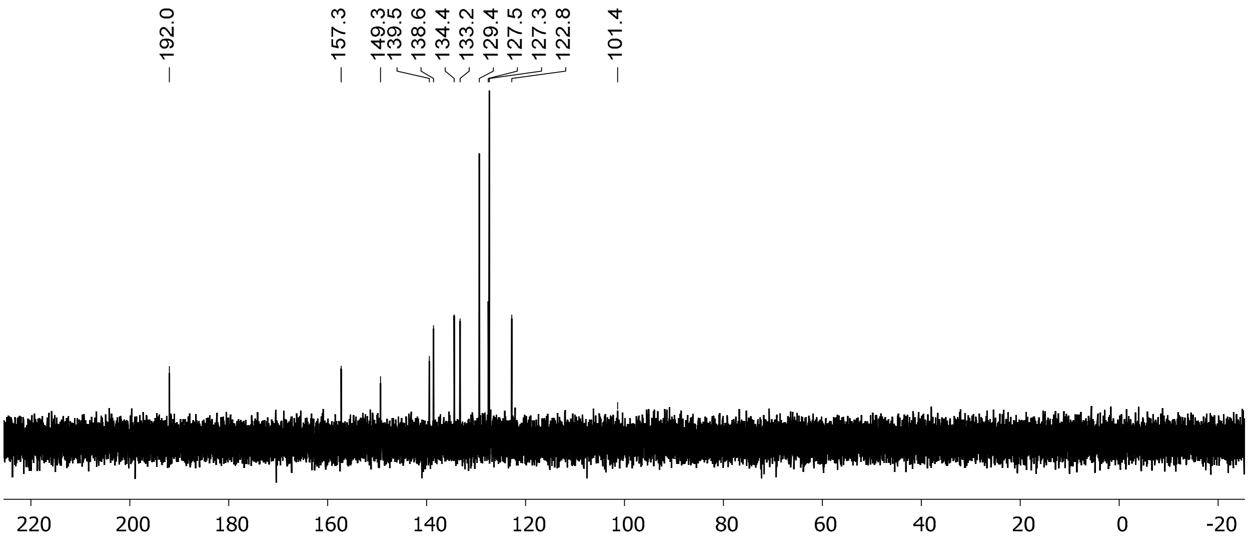

^{13}C NMR spectrum (151 MHz, D_2O) of **2e**

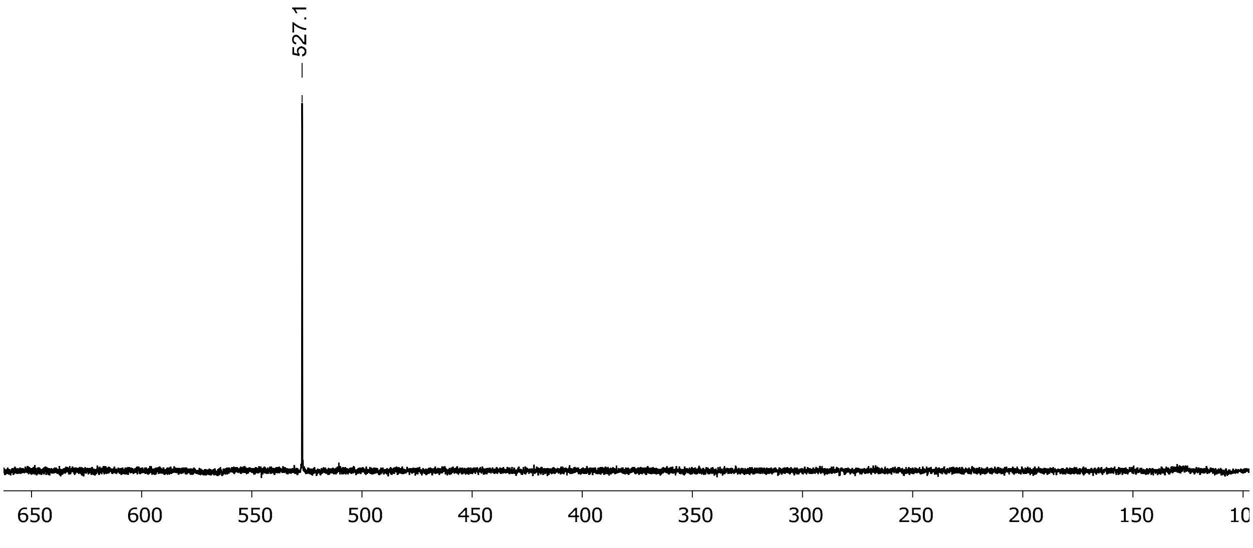

^{77}Se NMR spectrum (95 MHz, MeOH-*d*$_4$) of **2e**

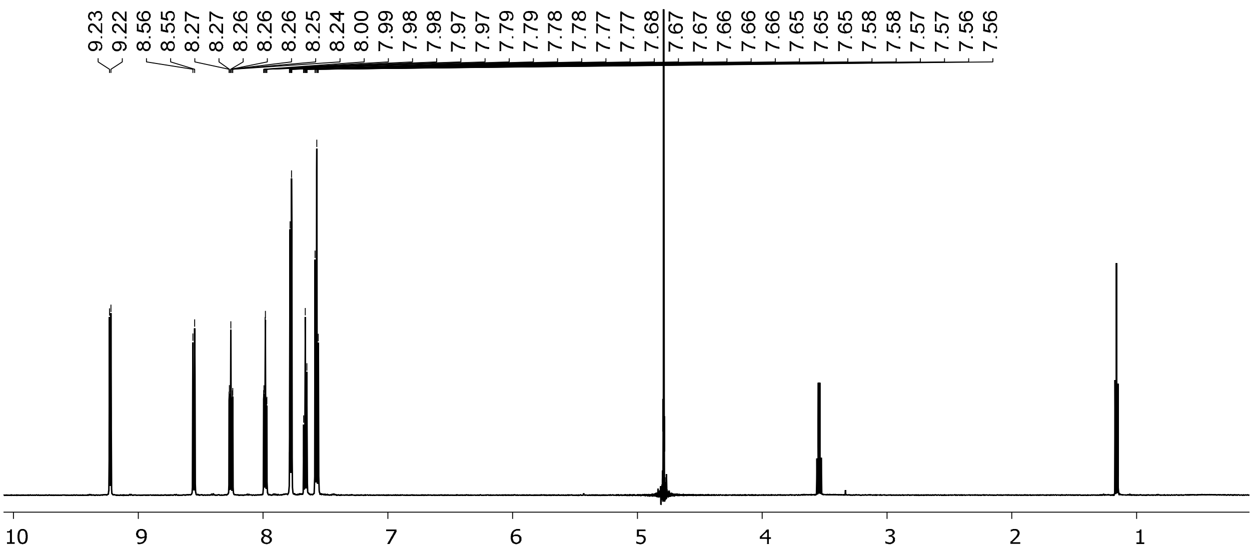

^1H NMR spectrum (700 MHz, D_2O) of **2f**

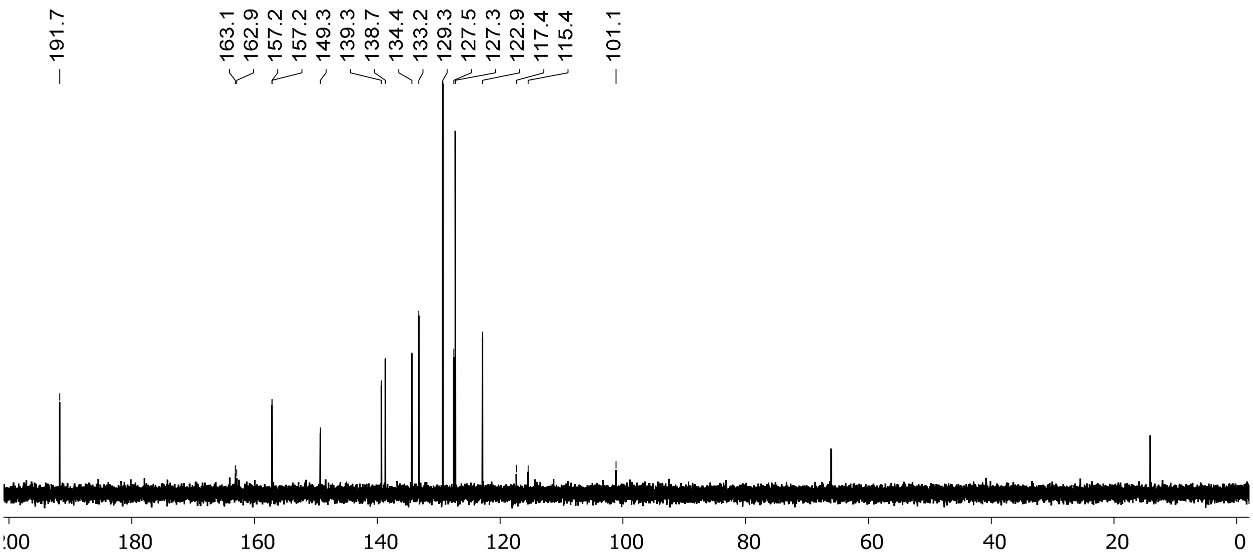

^{13}C NMR spectrum (151 MHz, D_2O) of **2f**

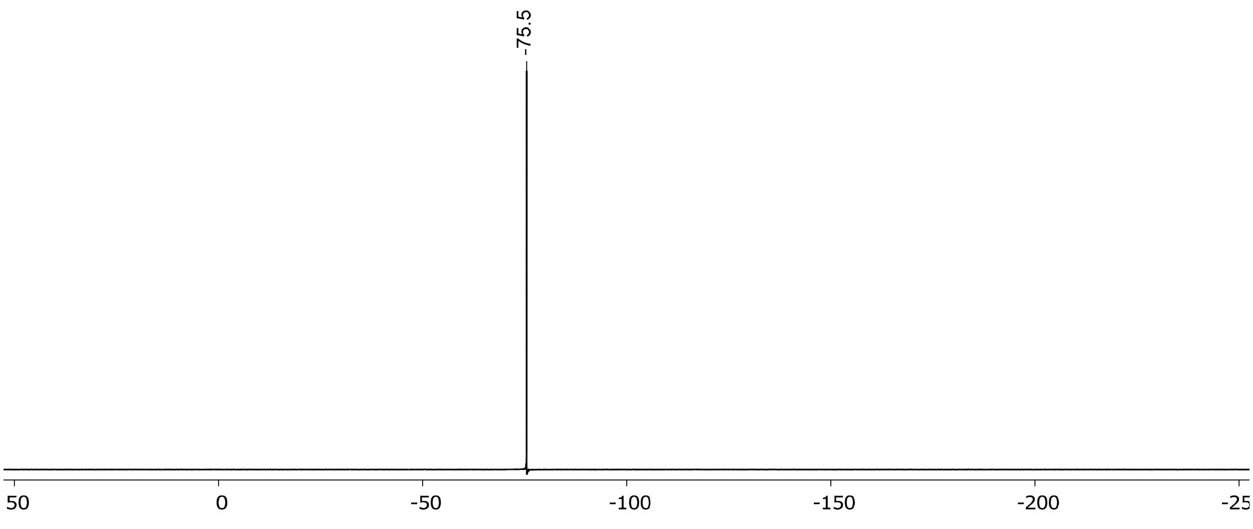

^{19}F NMR spectrum (565 MHz, D_2O) of **2f**

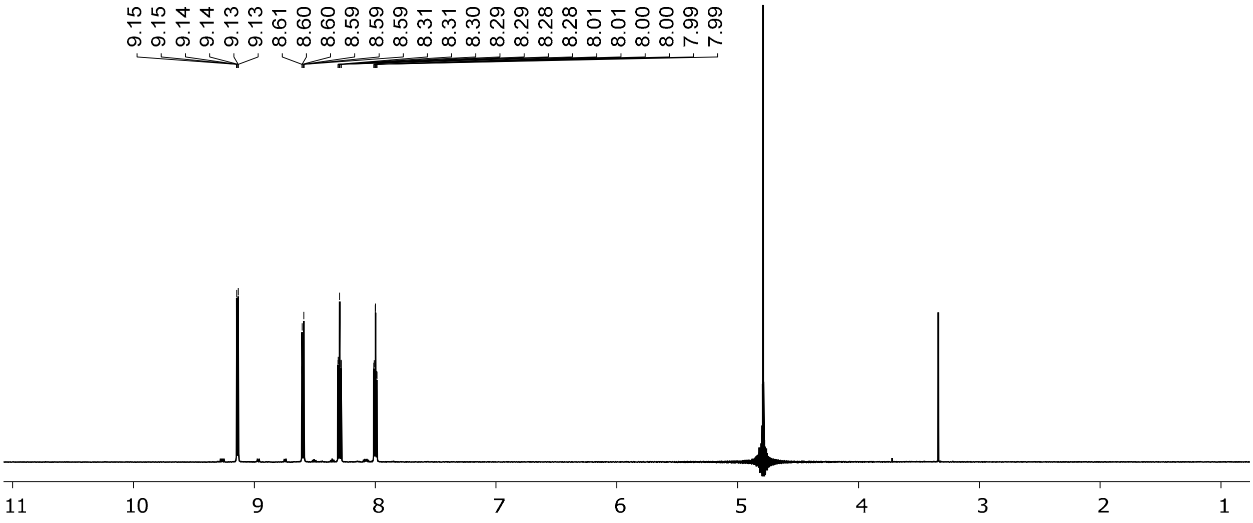

^{1}H NMR spectrum (700 MHz, D_2O) of **2g**

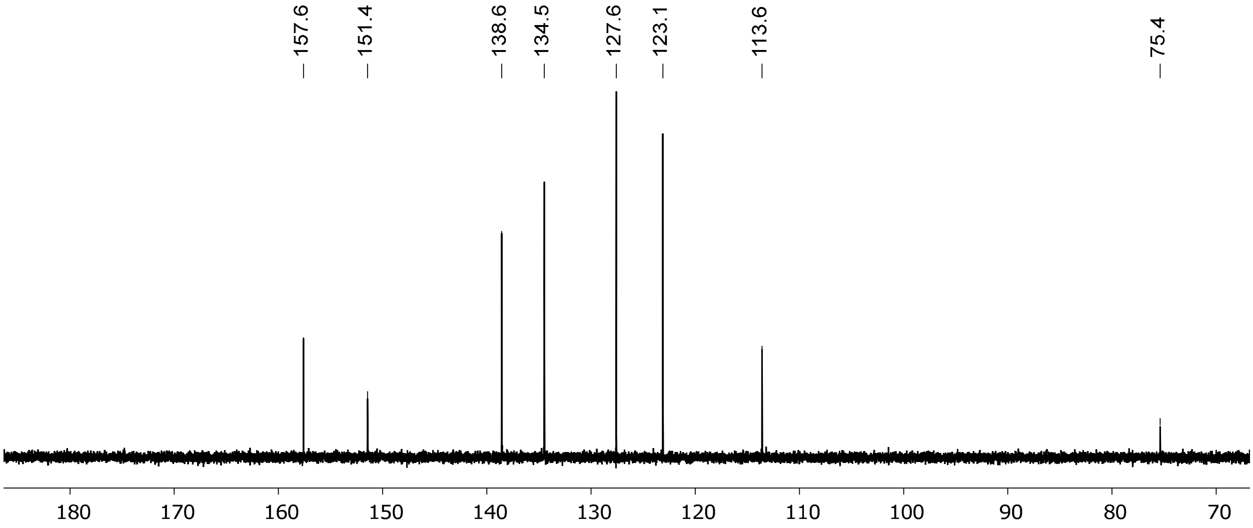

^{13}C NMR spectrum (176 MHz, D_2O) of **2g**

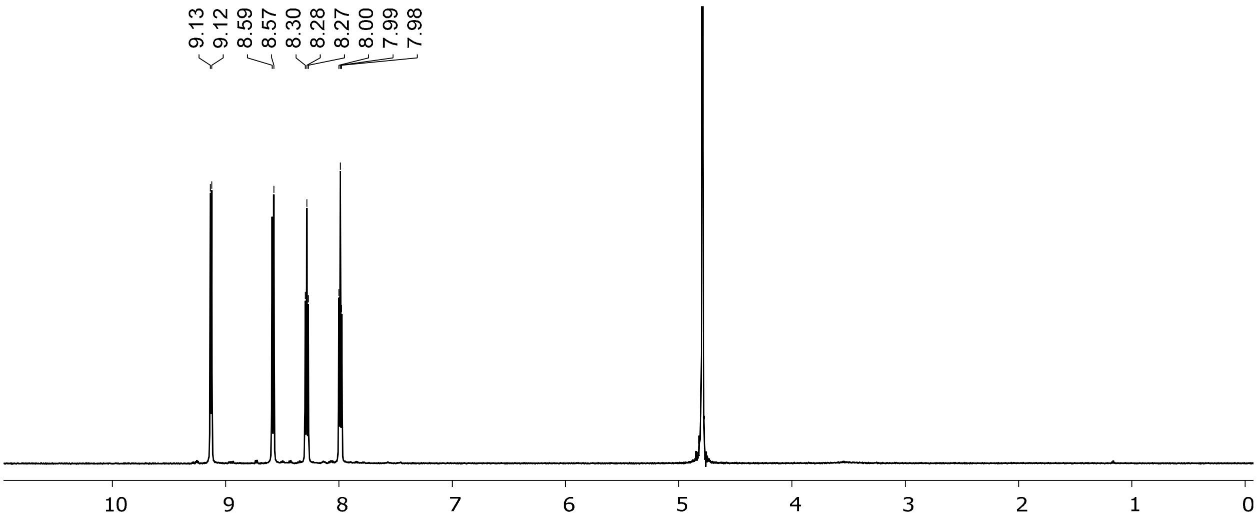

^{1}H NMR spectrum (600 MHz, D_2O) of **2h**

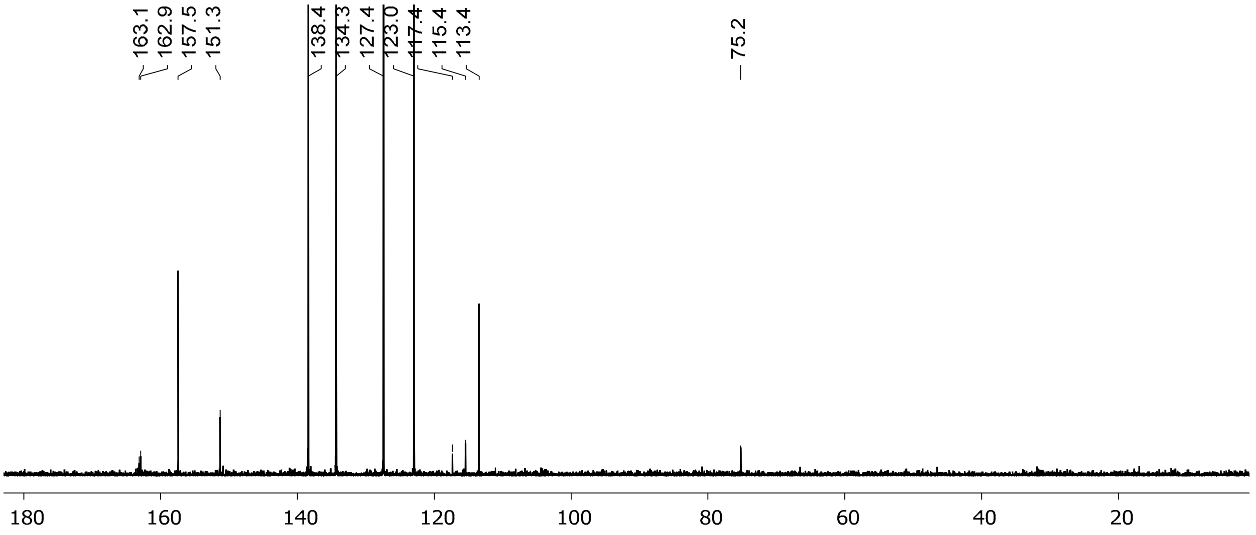

^{13}C NMR spectrum (151 MHz, D_2O) of **2h**

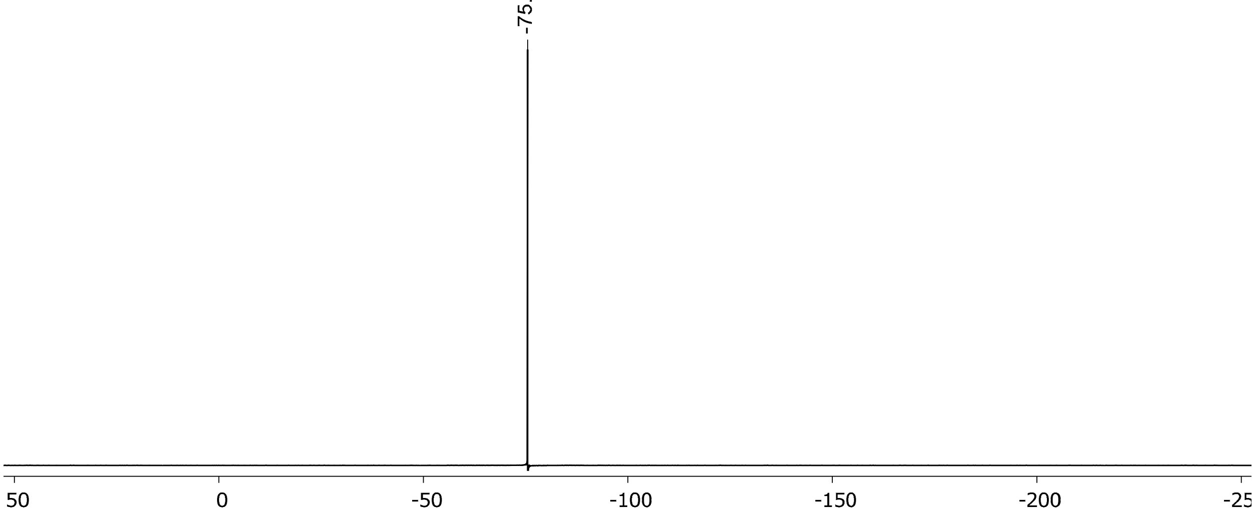

^{19}F NMR spectrum (565 MHz, D_2O) of **2h**

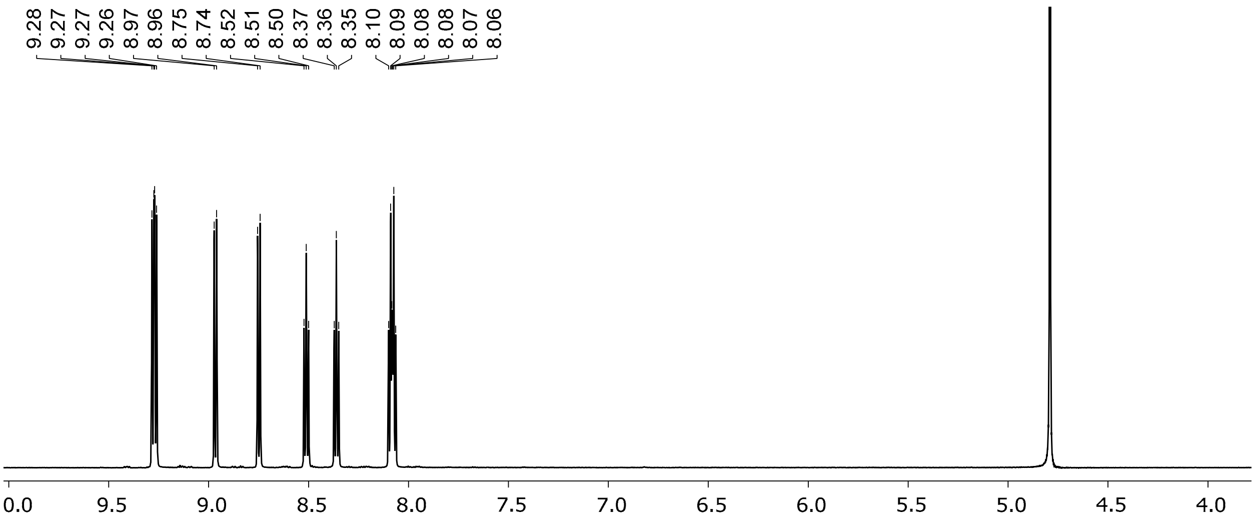

^{1}H NMR spectrum (700 MHz, D_2O) of **2i**

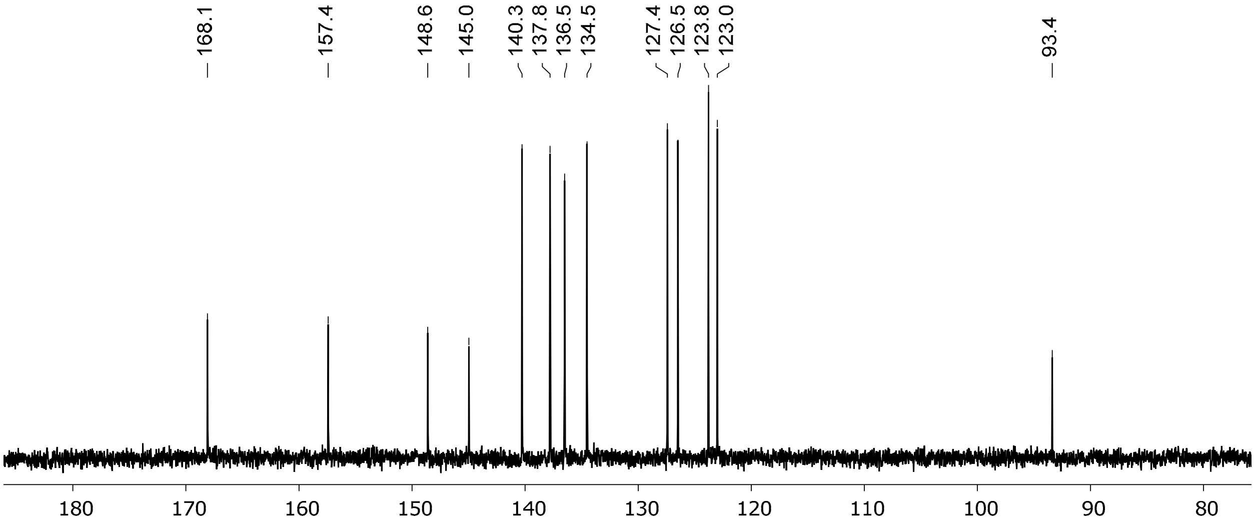

^{13}C NMR spectrum (176 MHz, D_2O) of **2i**

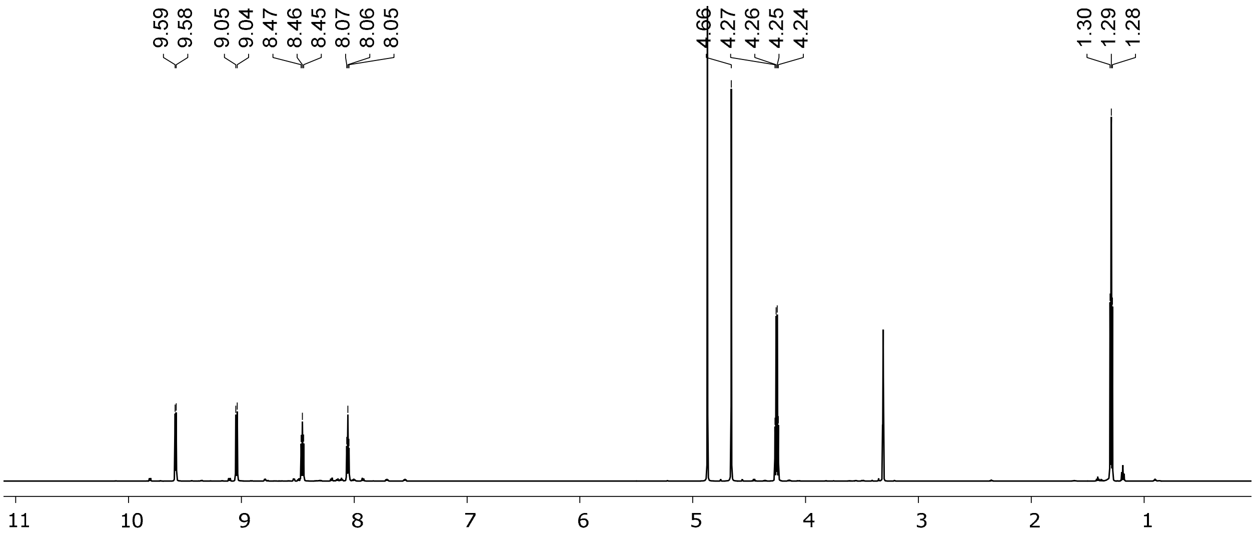

^{1}H NMR spectrum (700 MHz, MeOH-*d4*) of **3a**

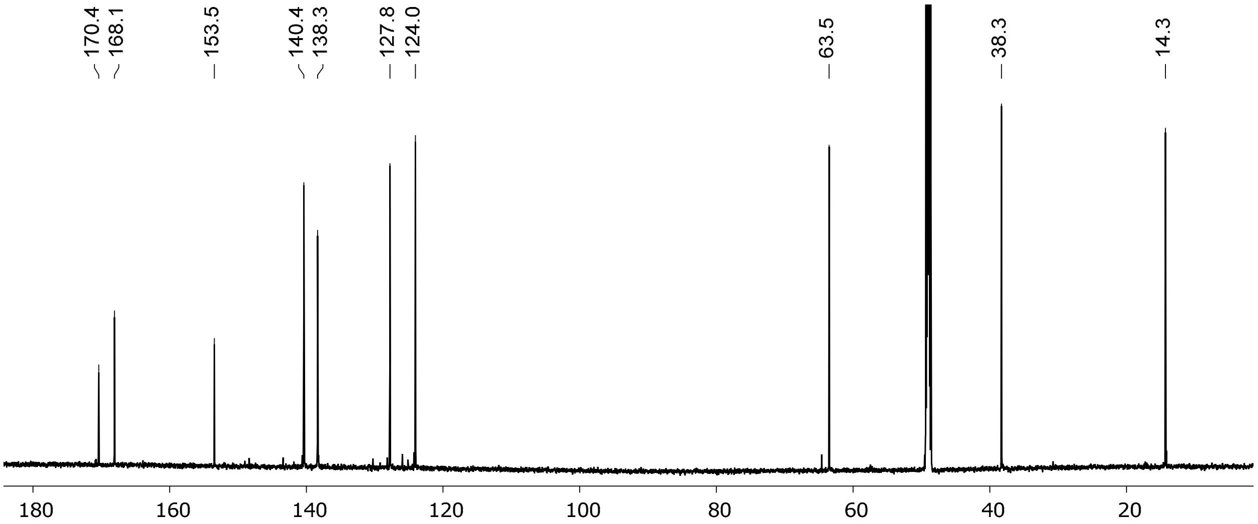

^{13}C NMR spectrum (176 MHz, MeOH-*d4*) of **3a**

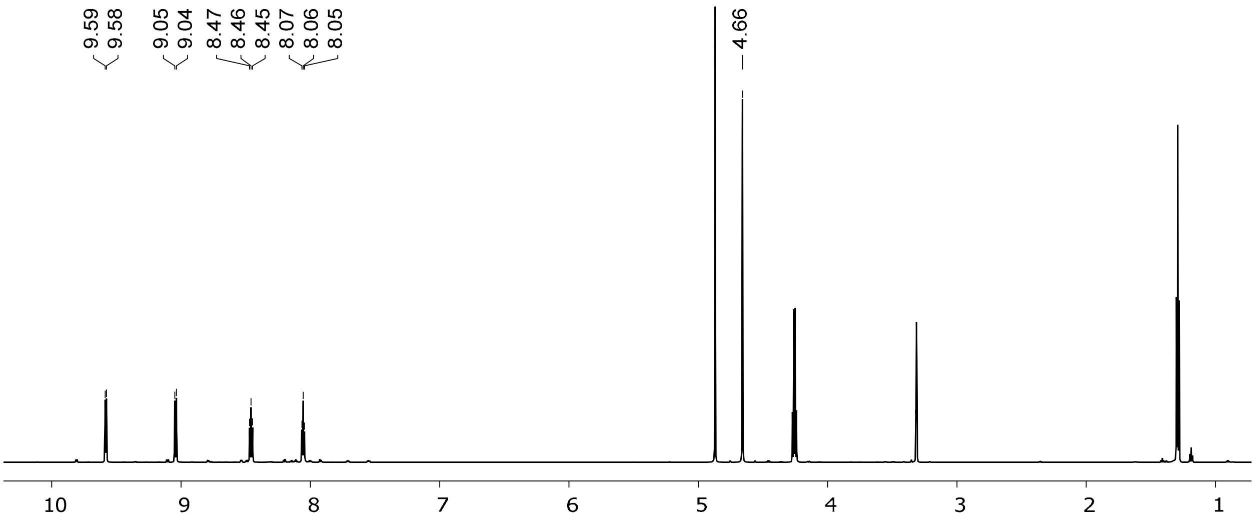

^{1}H NMR spectrum (700 MHz, MeOH-*d4*) of **3b**

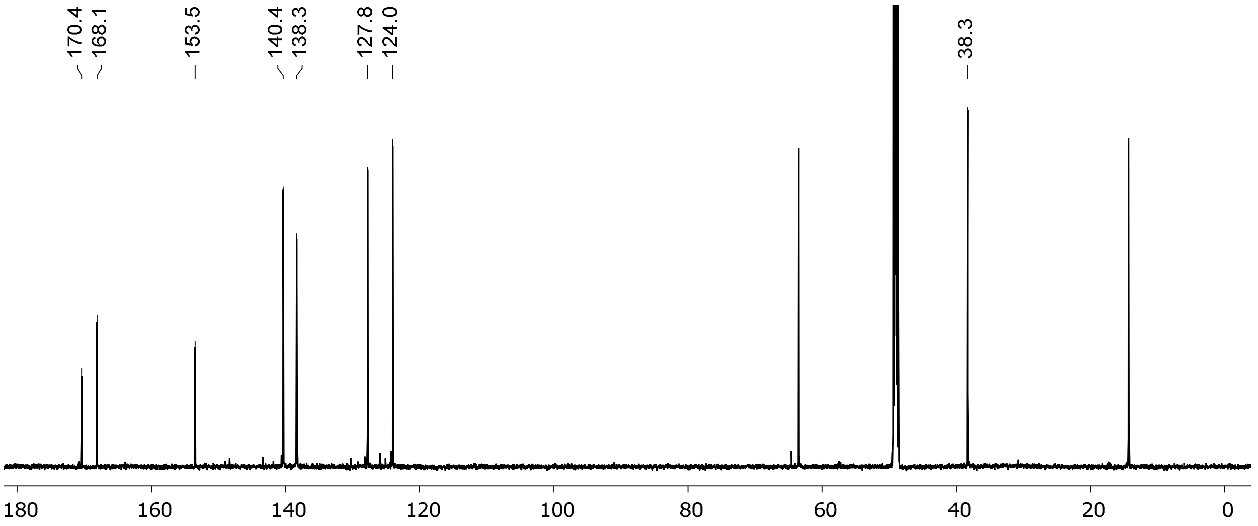

^{13}C NMR spectrum (176 MHz, MeOH-*d4*) of **3b**

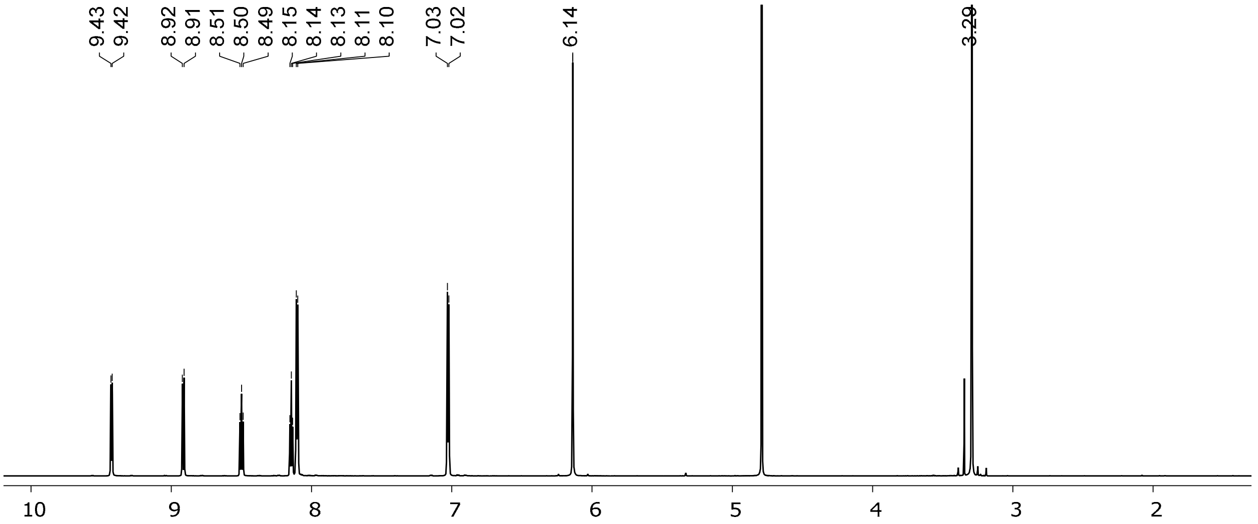

^{1}H NMR spectrum (700 MHz, D_2O) of **3d**

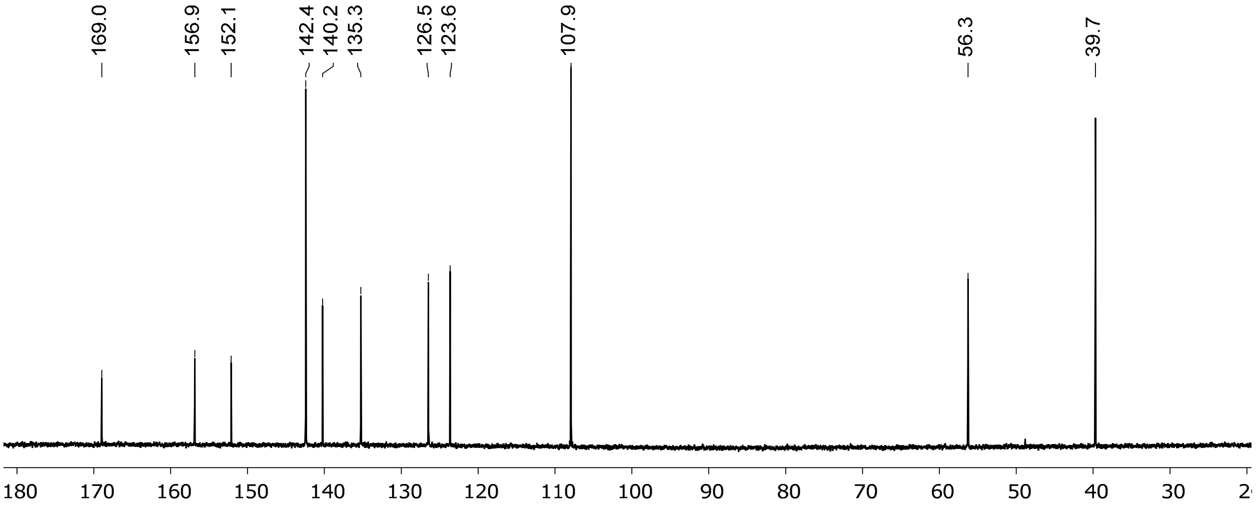

^{13}C NMR spectrum (176 MHz, D_2O) of **3d**

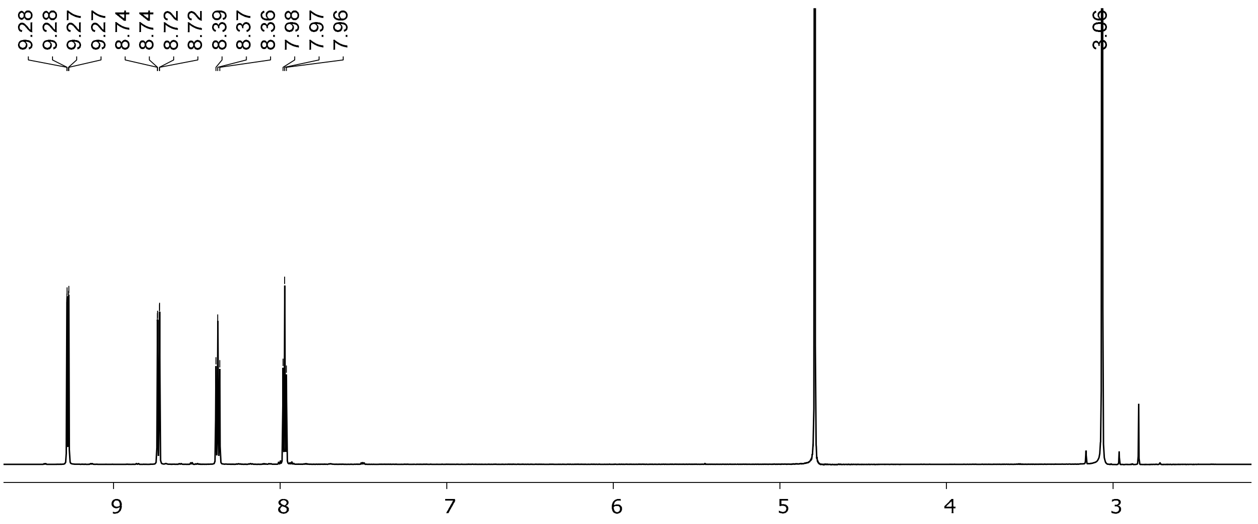

^{1}H NMR spectrum (700 MHz, D_2O) of **5**

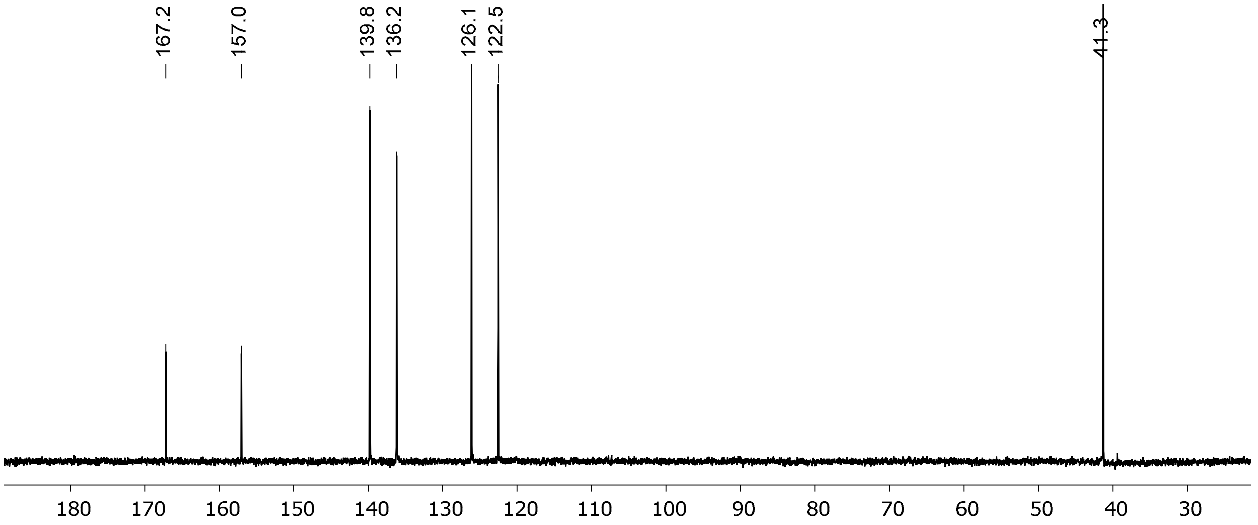

^{13}C NMR spectrum (176 MHz, D_2O) of **5**

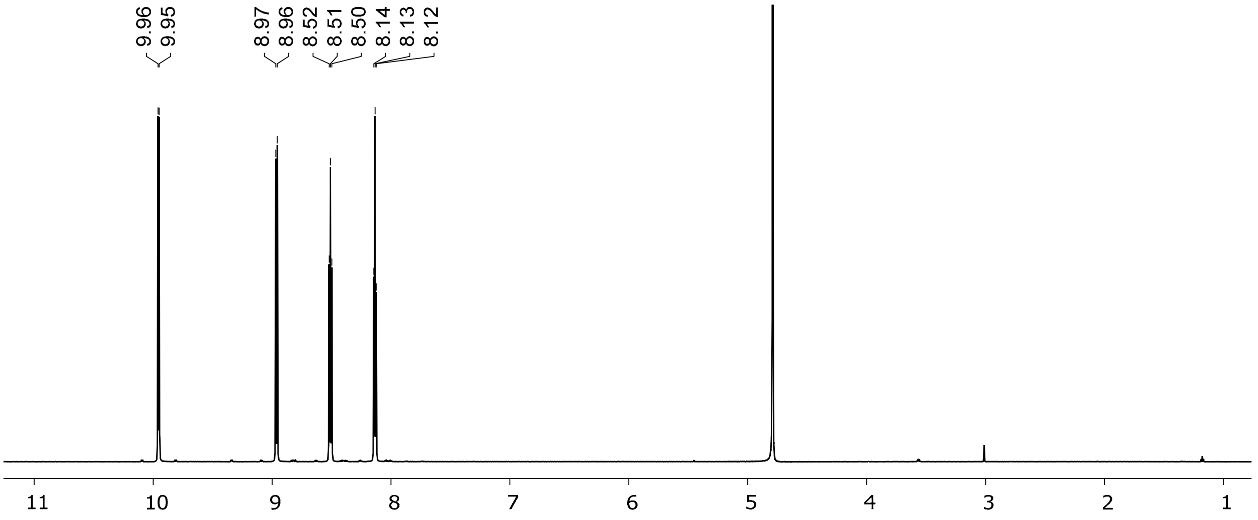

^{1}H NMR spectrum (700 MHz, D_2O) of **6**

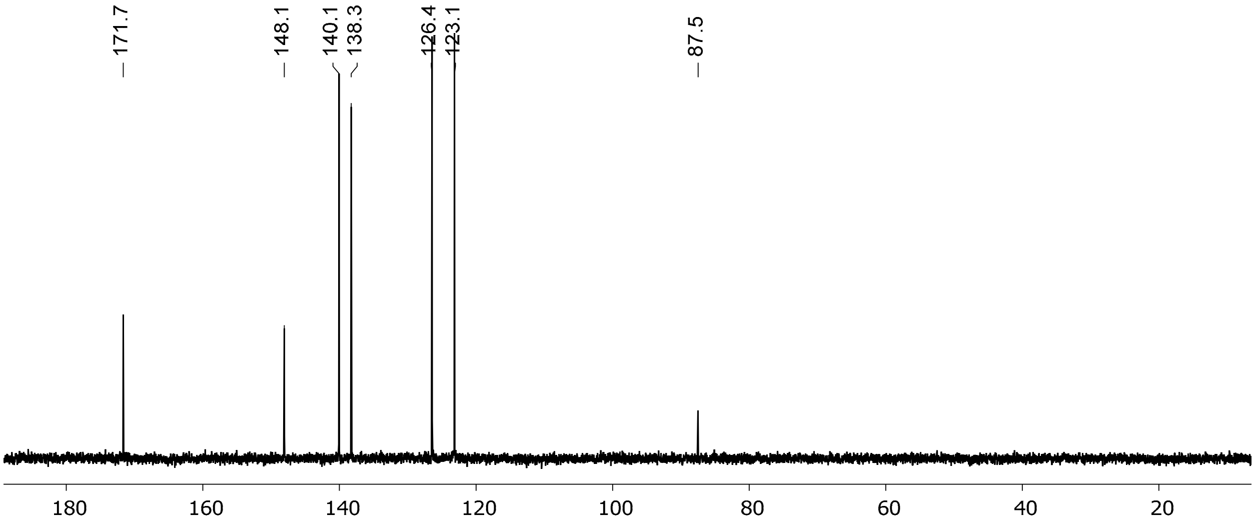

^{13}C NMR spectrum (176 MHz, D_2O) of **6**

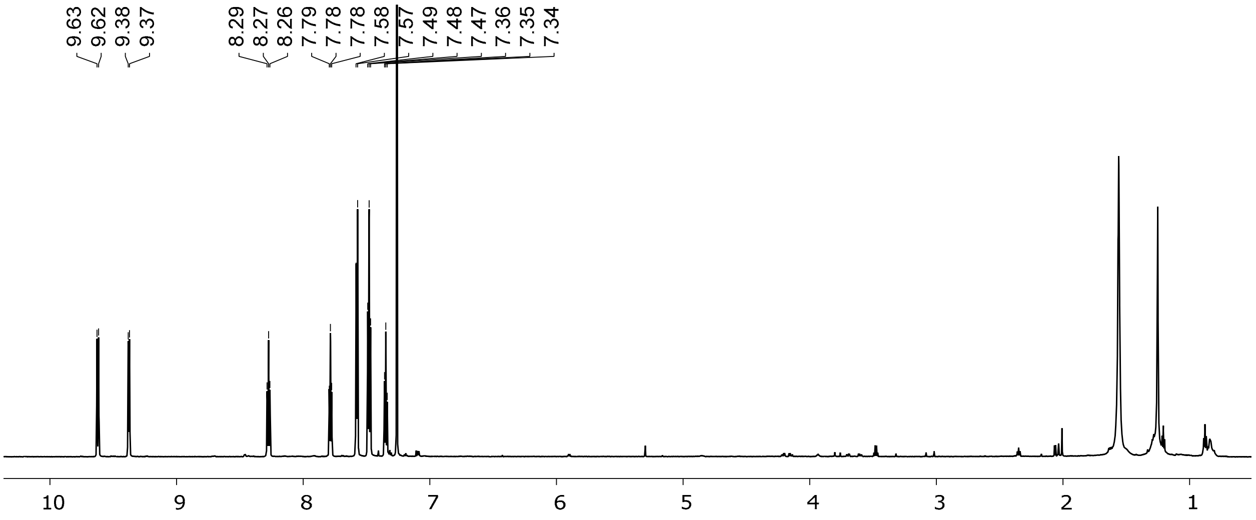

^{1}H NMR spectrum (700 MHz, $CDCl_3$) of **7**

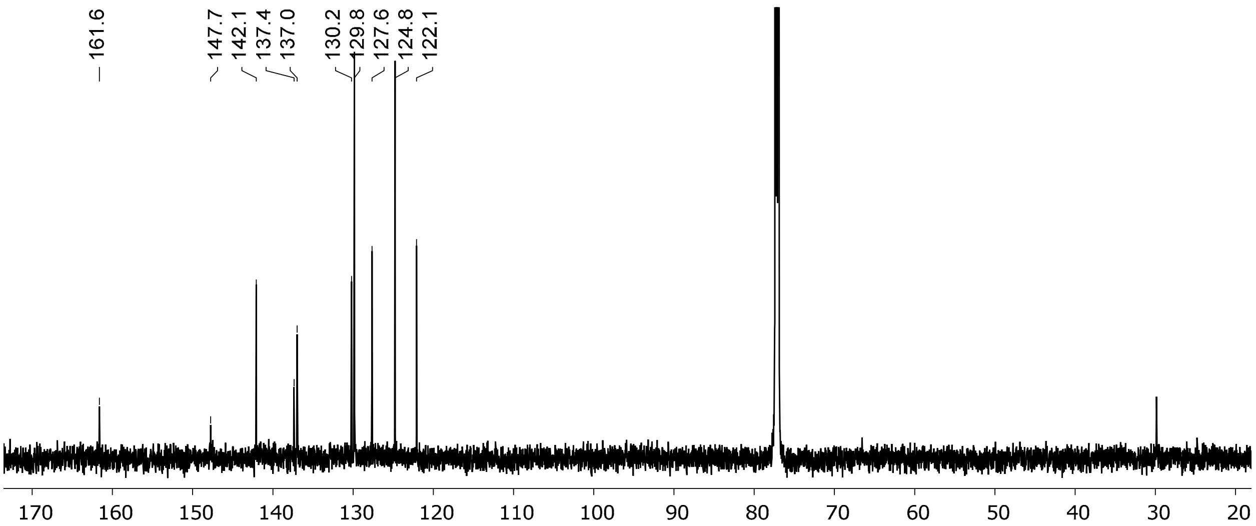

^{13}C NMR spectrum (176 MHz, $CDCl_3$) of **7**

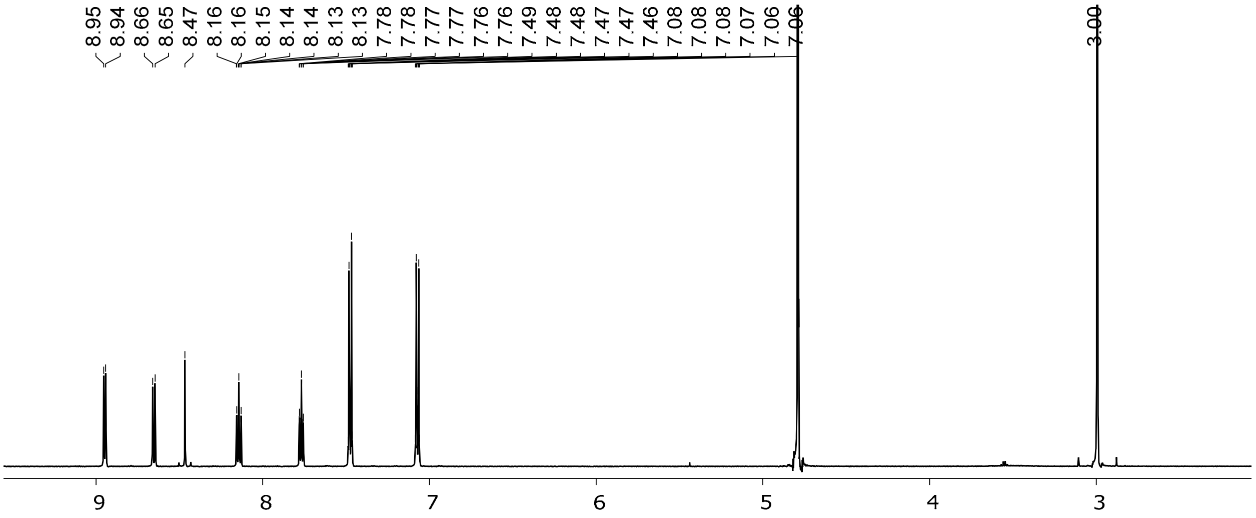

^{1}H NMR spectrum (600 MHz, D_2O) of **8**

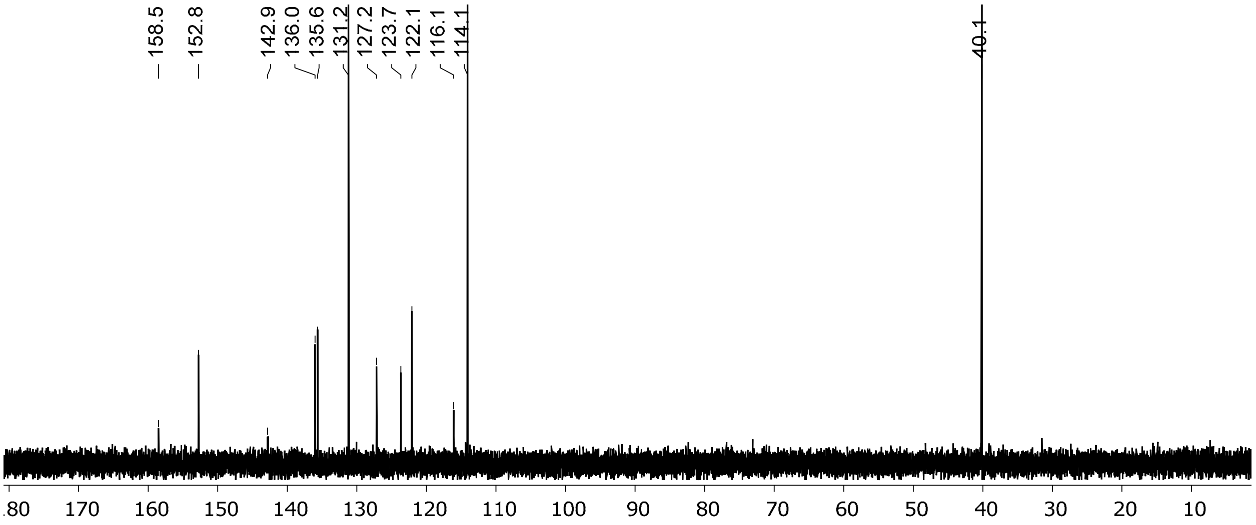

^{13}C NMR spectrum (151 MHz, D_2O) of **8**

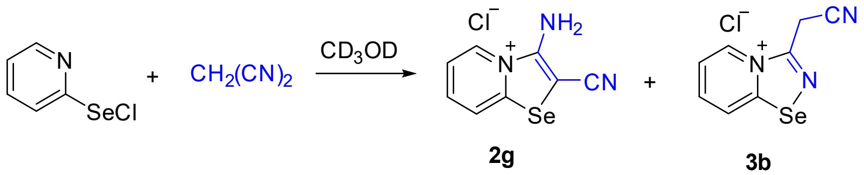

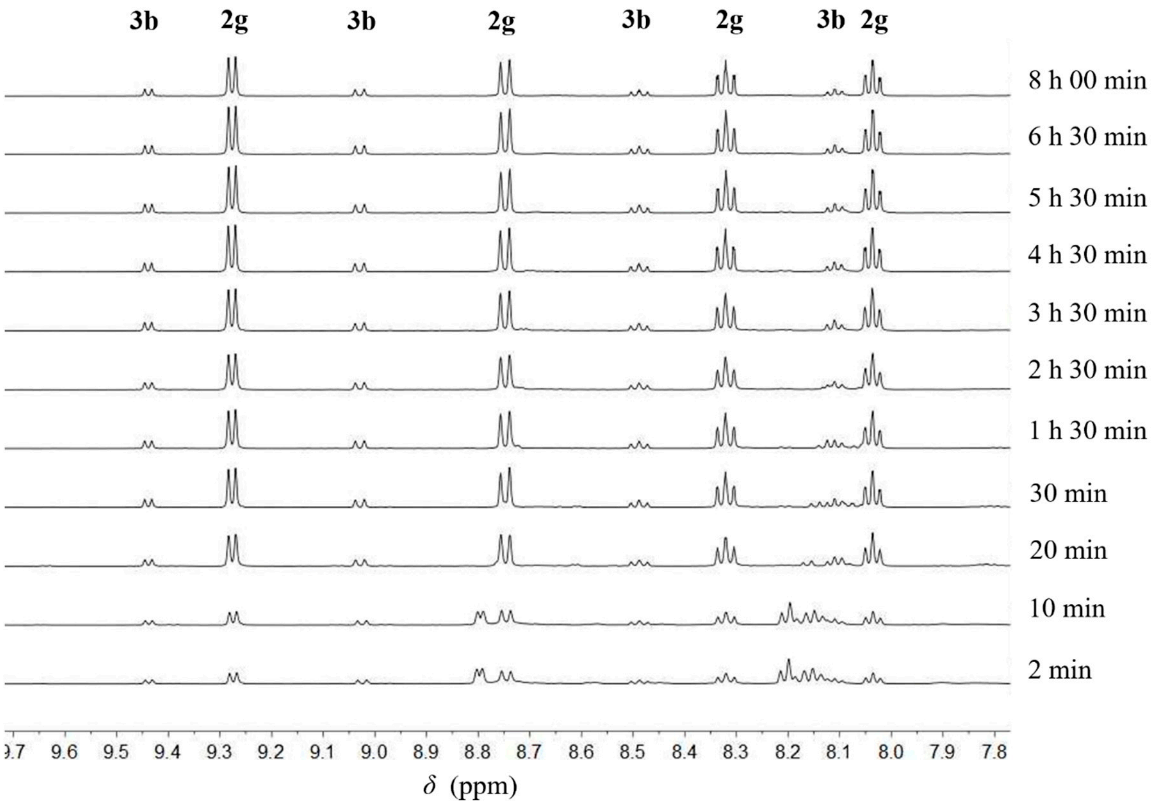

Figure S1. ^{1}H NMR (600 MHz, CD_3OD, room temperature) monitoring of the reaction between **1a** and malononitrile.

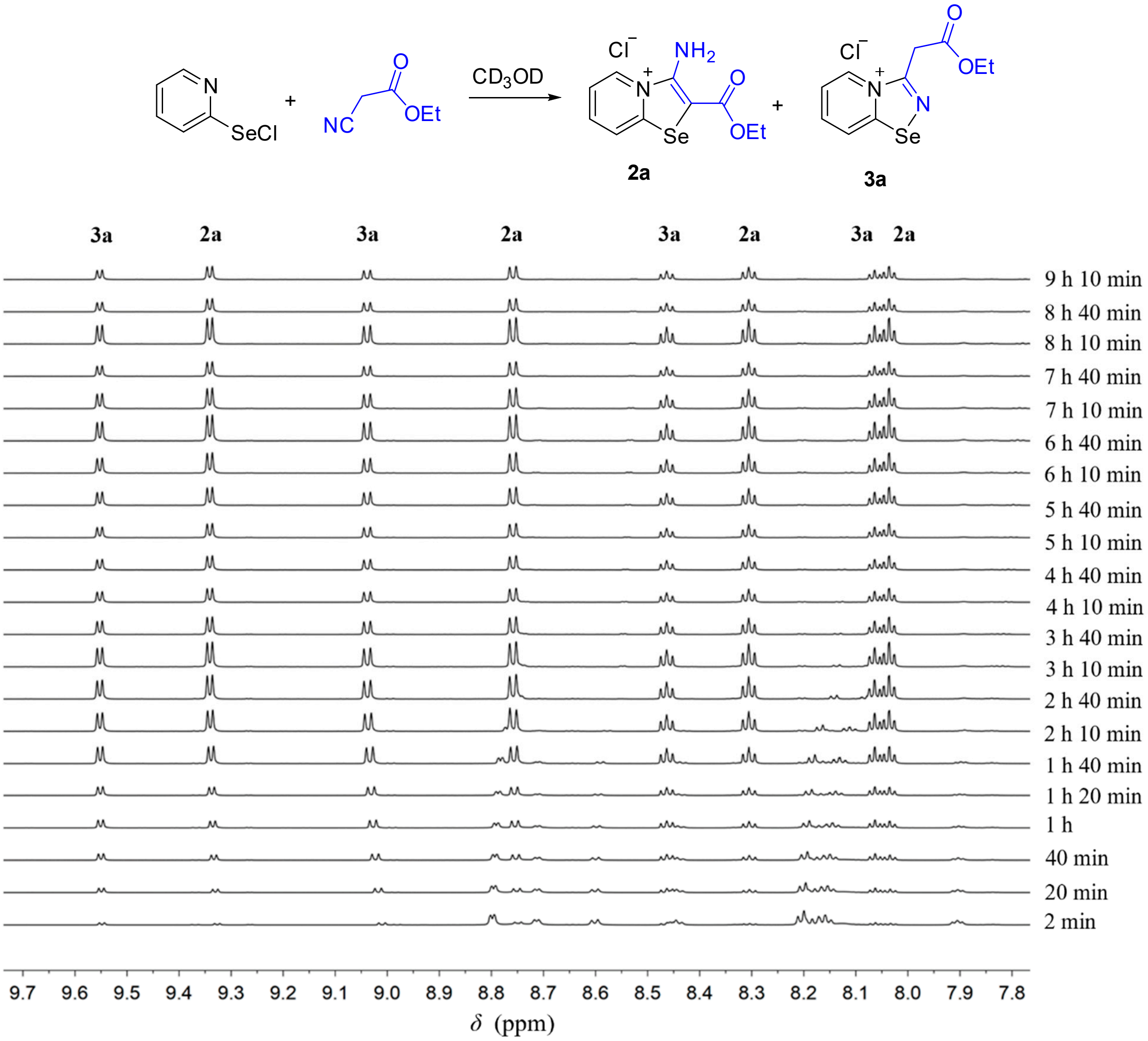

Figure S2. ^{1}H NMR (600 MHz, CD_3OD, room temperature) monitoring of the reaction between **1a** and ethylcyanoacetate.

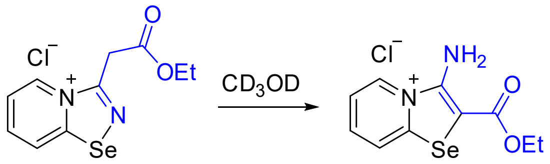

3a **2a**

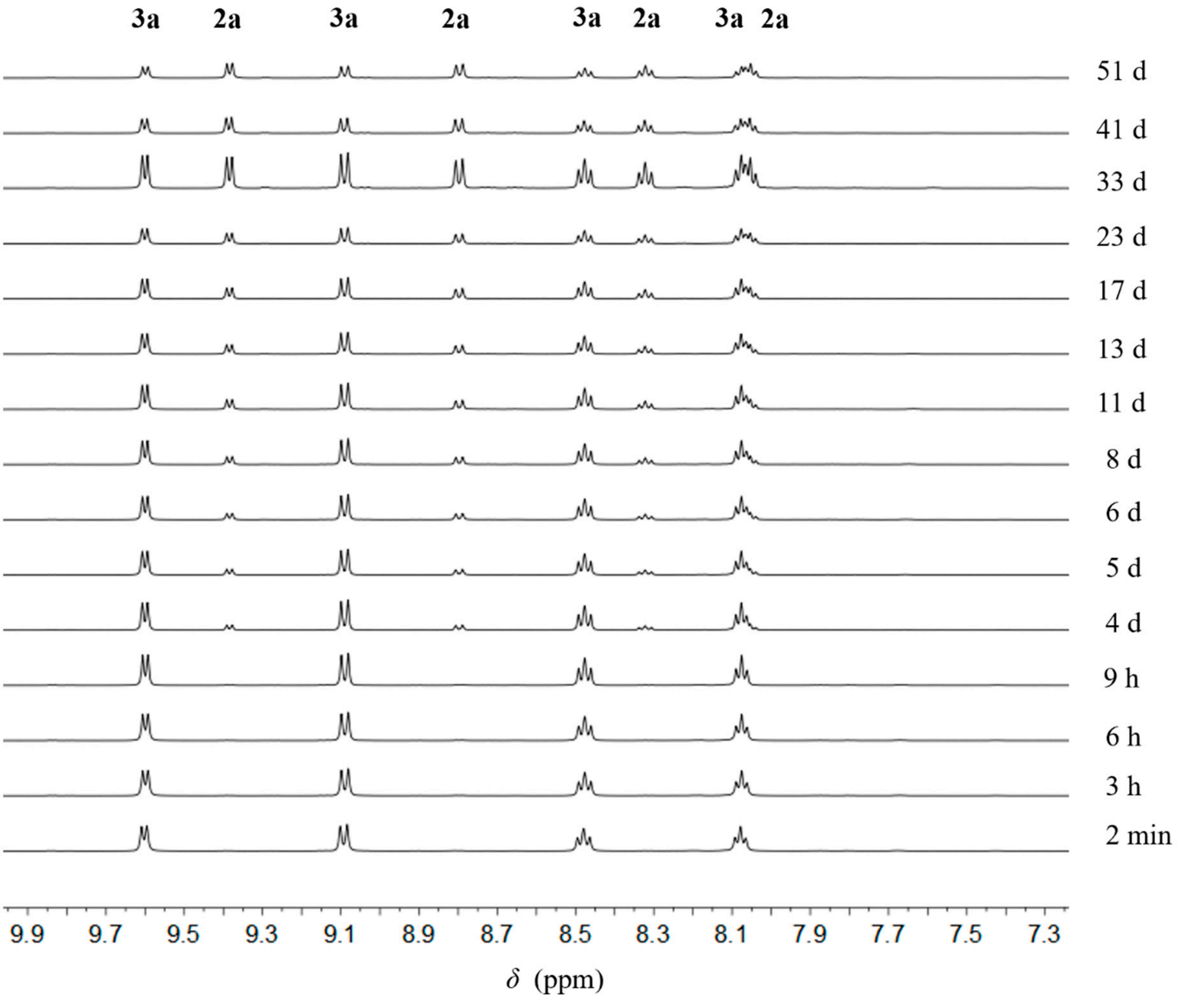

Figure S3. ^{1}H NMR (600 MHz, CD_3OD, room temperature) monitoring of the isomerization of **3a** into **2a**.
